# Polypeptide Sequence Effects on Virus Thermostability in Complex Coacervate Formulations

**DOI:** 10.1021/acs.biomac.6c00747

**Published:** 2026-08-07

**Authors:** Pratik U. Joshi, Claire Decker, Arvind Sathyavageeswaran, Xianci Zeng, Hong Liu, Milad Kheirvari, Ebenezer Tumban, Lynn Manchester, Idris Tohidian, Eduardo Barbieri, Sarah L. Perry, Caryn L. Heldt

**Affiliations:** † Department of Chemical Engineering, 3968 Michigan Technological University, Houghton, Michigan 49931, United States; ‡ Health Research Institute, Michigan Technological University, Houghton, Michigan 49931, United States; § Department of Chemical and Biomolecular Engineering, 14707 University of Massachusetts Amherst, Amherst, Massachusetts 01003, United States; ∥ Institute for Applied Life Sciences, University of Massachusetts Amherst, Amherst, Massachusetts 01003, United States; ⊥ Graduate Program in One Health Sciences, School of Veterinary Medicine, 6177 Texas Tech University, Lubbock, Texas 79409, United States

## Abstract

Current vaccine formulations heavily rely on cold chains to avoid degradation during transportation and storage. Vaccines typically degrade when exposed to temperatures outside the 2–8 °C range, leading to waste and logistical challenges, particularly in rural areas. This study investigates the ability of poly­(lysine)- and poly­(glutamate)-based peptide coacervates to improve the thermal stability of porcine parvovirus (PPV), a model nonenveloped viral vaccine. We hypothesized that both the length and specific amino acid sequence of the peptides forming the coacervates would influence the stability of PPV. Long polypeptides (400–800 mers) provided significant protection, slowing PPV inactivation at 60 °C for up to 7 days by as much as 4 logs (10,000-fold), whereas shorter 48-mer homopolypeptides offered limited stability. Modifying peptide sequences revealed that glutamate-glycine block copolypeptides at larger block sizes improved thermostability, while incorporating alanine residues into lysine block copolypeptides improved stabilization beyond that achieved with long homopolypeptides. The addition of sucrose in the formulations further improved thermostability, while trehalose showed minimal benefit. Although coacervation did not have a significant impact on viral infectivity, *in vivo* studies leveraging an alum adjuvant demonstrated that PPV released from a coacervate yielded lower antibody responses compared to native virus, indicating the presence of complicating interactions that potentially masked the immunogenic epitopes on the capsid surface or decreased the effectiveness of the adjuvant. Overall, this study showed that coacervate formulations can be adjusted to enhance virus thermal stability; however, further work is necessary to understand how such formulations can provide thermostability without altering the immune response necessary for a successful vaccine. Such design principles would enable the development of formulations that could decrease the vaccine cold-chain dependence and improve vaccine accessibility.

## Graphical abstract

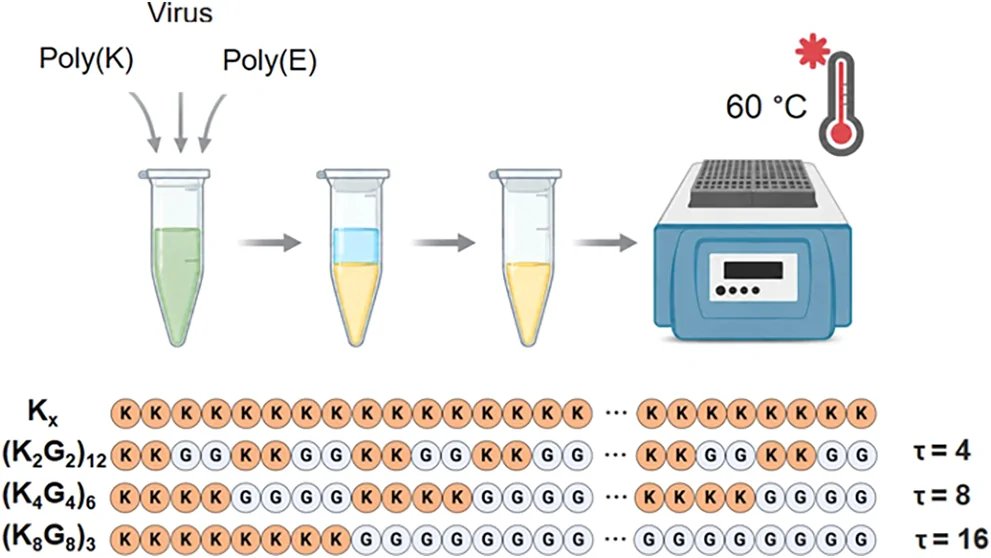

## Introduction

Vaccines are thermosensitive biological products and degrade rapidly if exposed to temperatures outside the recommended ranges. This necessitates a “cold chain,” a temperature-controlled supply chain from manufacturing to administration.^1^ Maintaining this chain involves challenging logistics,^2^ as well as issues such as insufficient storage,^3^ high refrigeration costs,^4^ and inadequate monitoring,^5^. which can lead to vaccine waste. These difficulties can hinder vaccine accessibility, even in high-income countries like the United States. For example, during the COVID-19 pandemic, rural residents faced significant logistical barriers that resulted in lower vaccination rates compared to urban residents.^6^ Developing thermostable vaccines could reduce the reliance on the cold chain, decrease costs, and improve vaccine accessibility.

Traditionally, vaccines are prepared either in a liquid or dry state. Liquid vaccine formulations, which are simple to manufacture and are ready to use, preserve the antigenic component by addition of stabilizing excipients.^7^ Dry formulations, on the other hand, prevent vaccine degradation by limiting the molecular mobility of the antigenic component via dehydration and the addition of drying-stabilizing excipients.^8^ Dry formulations typically achieve greater stability than liquid formulations, yet they require additional manufacturing steps and reconstitution before administration. Despite these strategies, most vaccines approved in the United States still require strict temperature handling that maintains the vaccine in the range of 2–8 °C.^9^

To overcome the inherent limitations of traditional formulations, a growing area of research is exploring novel material systems that can provide advanced protection for biological molecules. Some examples of strategies involve encapsulating the antigen in a cellulosic polymer^10^ or a biopolymer thin film.^11^ Other approaches explored bioinspired mineralization techniques to increase viral thermal stability. Silicification^12^ and calcification^13^ of enterovirus have been reported to increase its thermal stability. However, biomineralization is virus-specific, as the presence of nucleating peptides on the surface of the virus is necessary for the successful implementation of the biosilicification process. Overall, there is still a need to find better encapsulation methods to enhance the viral thermostability.

Complex coacervation has emerged as a promising strategy for improving thermal stability of biological molecules by creating a unique protective microenvironment. Complex coacervation is a liquid–liquid phase separation phenomenon that occurs when oppositely charged macro-ions are mixed in solution and undergo associative phase separation into a dense, macro-ion-rich phase called the coacervate, and a dilute phase called the supernatant.^14^ The associative nature of coacervation facilitates the incorporation of proteins, viruses, and other biomolecules at high concentrations (*i.e*., coacervation is not merely an encapsulation strategy, as the biomolecule is an active participant in the formation of the complex). Furthermore, the dense nature of the coacervate phase mimics the crowded interior of living cells and can help prevent protein unfolding and aggregation.^15–19^ For instance, bovine serum albumin (BSA) maintained its secondary structure within a coacervate and after disassembly^20^ as well as following 12 weeks of storage at room temperature.^17^ The utility of coacervation also extends to improving the thermostability of larger biomolecules, including viruses,^16^ which are often the key components in vaccines. Biological condensates, which are in many ways analogous to coacervates, have been shown to play a critical role in the assembly of a number of viruses,^21–24^ and coacervates have even been considered as possible compartments that could help concentrate and stabilize primitive macromolecules associated with the origin of life.^25–31^ The formation of synthetic coacervates also has the benefit of being highly tunable, allowing for variation in factors like the charge ratio of the macro-ions, ionic strength, pH, as well as the size and charge density of macro-ions.^29,32–34^

In addition to encapsulation strategies, small-molecule excipients such as sugars are widely used to thermostabilize proteins^30,35–37^ and in vaccine formulations.^38–40^ Increased protein thermal stability is often explained by indirect interactions where sugar molecules change the interactions among protein, water, and the sugar molecule itself. This often results in sugar molecules being preferentially excluded from the local domain of the protein, creating a denser, more structured water network surrounding the protein, protecting the folded state.^41,42^ Disaccharides are known to better preserve proteins compared to monosaccharides, as the degree of exclusion usually increases with size.^43^ Drying can also increase protein stability by reducing the amount of water in the formulation.^44^ However, it is an open question whether the beneficial effects of sugars would work synergistically with coacervation strategies because of the dense nature of the coacervate phase.

Here, we utilize polypeptide-based complex coacervates because of the potential to specifically recapitulate the high concentrations of protein present in the cellular milieu. Furthermore, we can control the sequence of amino acids within a polypeptide in order to tune the material environment of the coacervate to optimize biomolecule uptake and stability,^29,32,45–47^ Previously, our group has shown that incorporation of porcine parvovirus (PPV), a nonenveloped virus, into a polypeptide complex resulted in superior thermal stability during accelerating aging experiments at 60 °C compared to free PPV.^16^ Furthermore, we demonstrated that the hydrophobicity and charge patterning of the coacervating peptides influenced virus uptake into the coacervate.^32^ Here, we demonstrate how polypeptide coacervate systems with sequences of charge and hydrophobicity can be leveraged to enhance the thermostability of PPV formulations. We also explore how small-molecule excipients, like sugars, provide synergies with coacervates to improve virus stability. Finally, we explore the potential for using disassembled coacervate formulations in tandem with an alum adjuvant to generate an immune response from vaccine-like injections.

### Materials and Methods

### Materials

The zwitterionic buffer 4-(2-hydroxyethyl)-1-piperazineethanesulfonic acid (HEPES, ≥99%), anhydrous ethyl ether, dichloromethane (DCM), *N,N*-dimethylformamide (DMF), acetonitrile, hydrochloric acid (HCl), sodium chloride, and sodium hydroxide (NaOH) were purchased from Fisher Scientific, Hampton, NH. Piperidine, ethyl­(hydroxyiminio)­cyanoacetate (Oxyma), trifluoroacetic acid (TFA), Fmoc-l-Lys­(Boc)–OH, Fmoc-d-Lys­(Boc)–OH, Fmoc-l-Glu­(OtBu)–OH, Fmoc-d-Glu­(OtBu)–OH, Fmoc-Gly-OH, Fmoc-l-Ala-OH, Fmoc-l-Leu-OH, and α-cyano-4-hydroxycinnamic acid were purchased from Sigma-Aldrich, St. Louis, MO. Rink amide MBHA resin with a loading capacity of 0.643 mmol/g was purchased from P3 Biosystems, Louisville, KY. *N,N*-Diisopropylcarbodiimide (DIC, 99%) was purchased from Acros Organics, Waltham, MA. Triisopropylsilane (TIPS) was purchased from Chem-Impex International, Wood Dale, IL. Sucrose was purchased from MilliporeSigma, Burlington, MA. All chemicals were used as received, without further purification.

Poly­(l-lysine trifluoroacetate) and racemic poly­(d,l-glutamate sodium salt) with chain lengths of 400 and 800 (K_400_, K_800_, E_400_, E_800_) were purchased from Alamanda Polymers, Huntsville, AL, and used without further purification. Shorter polymers of poly­(l-lysine trifluoroacetate) and poly­(d,l-glutamate sodium salt) with a degree of polymerization of *N* = 48 (K_48_, E_48_) and copolymers of lysine or glutamate with glycine, alanine, and leucine were made in-house via solid-phase synthesis. A summary of these polymers is given in Table S1.

Eagle’s minimum essential medium (EMEM), phosphate-buffered saline (PBS, pH 7.2), penicillin–streptomycin (pen–strep), trypsin containing EDTA, and fetal bovine serum (FBS, USDA approved) were purchased from Life Technologies, Carlsbad, CA. 2-(3,5-diphenyltetrazol-2-ium-2-yl)-4,5-dimethyl-1,3thiazolebromide (98%, MTT) was purchased from Alfa Aesar, Haverhill, MA, and sodium dodecyl sulfate (SDS, BioReagent, ≥98.5%) was purchased from Sigma-Aldrich. Aqueous solutions were made in deionized water obtained from a Milli-Q water purification system (MilliporeSigma) or a Nanopure water system (Thermo Fisher, Waltham, MA) at a resistivity of >18.2 MΩ cm.

### Cell Culture and Cell Toxicity

Porcine kidney cells (PK-13, ATCC CRL-6489) were cultured in EMEM supplemented with 10%v/v FBS and 1%v/v pen-strep as previously described.^32^ Cells were incubated at 37 °C, 5% CO_2_, and 100% relative humidity.

Coacervate toxicity was tested in Vero cells (ATCC, CCL-81) grown as previously described.^48^ Vero cells at 70% confluency had a 1:5 dilution of coacervate sample added and were serially diluted 1:5. After 24 h, 5 mg/mL of MTT in 1× pH 7.2 PBS was added to each well. After 4 h, 10 w/v% aqueous SDS solution with 0.01 M hydrochloric acid (HCl) was added to each well. The absorbance at 550 nm was measured with a Synergy Mx microplate reader from BioTek (Winooski, VT) the next day.

### Virus Propagation, Titration, and Purification

Porcine parvovirus strain NADL-2 (PPV) was propagated in PK-13 cells using a previously established method.^49^ After three freeze–thaw cycles, the cell lysate was clarified by centrifugation at 4500 rpm (3850*g*) at 4 °C for 15 min in an ST16R centrifuge with a TX-400 swinging-bucket rotor (Thermo Scientific, Waltham, MA). Unless otherwise noted, the virus-containing supernatant was stored at −80 °C and thawed prior to use.

The titer of the viruses was found by the MTT colorimetric cell viability assay, which determines the dilution of the virus that shows a cytopathic effect in 50% of the cells.^50^ Briefly, PK-13 cells were seeded at a density of 8 × 10^4^ cells/mL in 96-well plates and incubated overnight. The next day, the cells were infected with a 1:5 serial dilution of viral samples. After 6 days, MTT was added, as described in the Cell Culture and Cell Toxicity section. The 50% viral infectious dose was determined in units of MTT_50_/mL.

PPV was purified and concentrated prior to animal studies by buffer exchanging the clarified stock twice with 20 mM HEPES in an Amicon 100 kDa spin filter (MilliporeSigma, Burlington, MA). After the 2 buffer exchange steps, the sample was concentrated 10x.

### Peptide Synthesis and Characterization

Polypeptides with *N* = 48 were prepared using standard Fmoc-based solid-phase synthesis on a Liberty Blue automated microwave peptide synthesizer from CEM, Ltd. using Rink amide MBHA resin at a 0.1 mmol scale. 20% piperidine in DMF was used for Fmoc deprotection. 0.5 M DIC and 1 M Oxyma in DMF were used as activator and base, respectively. Side chain deprotection and cleavage of the polypeptide from the resin were performed using TFA, Milli-Q water, and TIPS in a volumetric ratio of 95/2.5/2.5 for 3 h at room temperature. Anhydrous ethyl ether at −80 °C was used to precipitate the crude polypeptide. The solution was then centrifuged (Thermo Scientific Sorvall Legend X1R Centrifuge) at 3500 rpm (1739*g*) for 5 min at 25 °C, the ether was poured out, and the peptide was lyophilized (Labconco FreeZone Freeze-Dryer). Characterization of the final polypeptides was performed using a Bruker UltrafleXtreme matrix-assisted laser desorption/ionization time-of-flight mass spectrometer (MALDI-TOF). Samples of the peptide were mixed with different ratios of a matrix consisting of 20 mg/mL in a 1:1 mixture of water and acetonitrile with 0.01% TFA. The MALDI results can be found in Figure S1a–j. Amino acids with alternating chirality were used in either the polycation K_48_ or the polyanions E_48_, E_400_, and E_800_ to prevent backbone hydrogen bonding.^51–53^

### Preparation of Stock Solutions

The HEPES buffer was prepared at a concentration of 400 mM and adjusted to pH 8.00 ± 0.03 using 1 M HCl and 1 M NaOH, as the stock buffer solution. The polypeptide solutions were prepared at a concentration of 10 mM based on a total ionizable monomer basis in a 10 mM HEPES buffer and then adjusted to pH 8.00 ± 0.03. 2 M sucrose and 2 M NaCl solutions were prepared without pH adjustment.

### Coacervate-Virus Sample Preparation

As shown in Figure 1a, to incorporate PPV in a complex coacervate, clarified viral stock was added to the diluted stock buffer solution, followed by the addition of polycation. The sample was vortexed for 5 s before the polyanion was added to it and then vortexed for another 5 s. The total sample volume was 240 μL. The charge fraction was adjusted by changing the ratio of polycation and polyanion introduced to the sample while keeping the total polypeptide concentration constant at 7 mM on an ionizable monomer basis.

**Figure 1:**
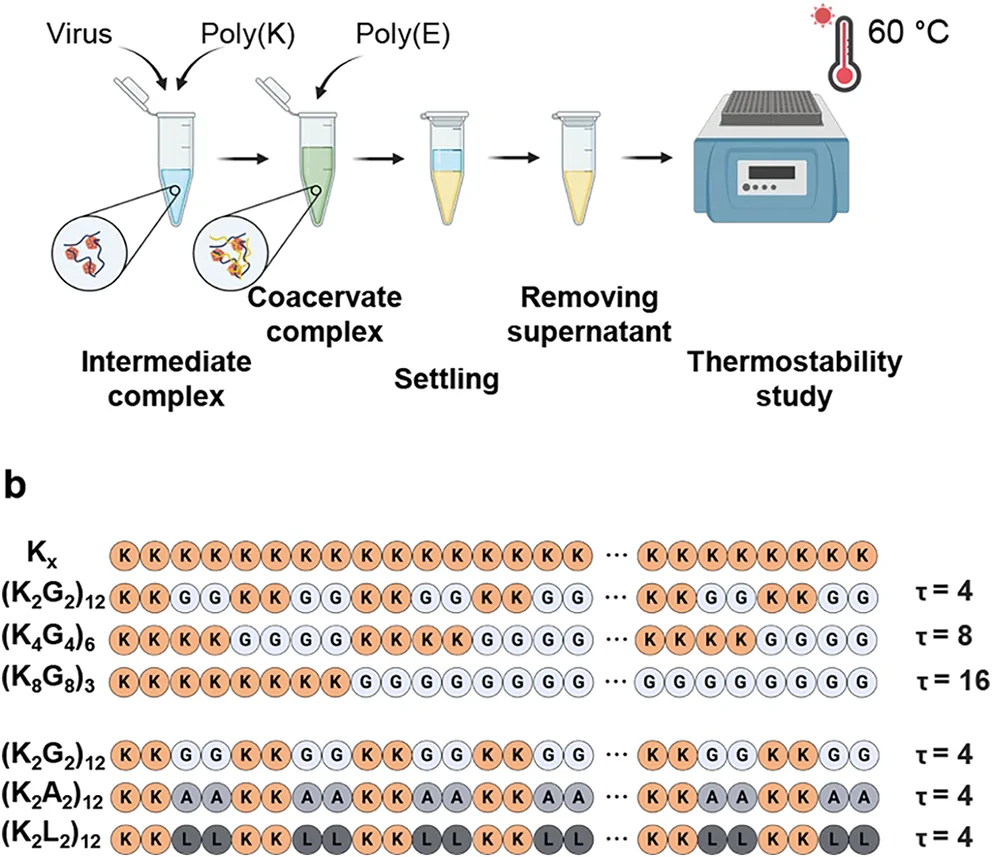
(a) Coacervation and thermostability workflow. (b) Examples of different charge densities and hydrophobicity of polycation containing positively charged lysine (K). The charge pattern is defined by introducing neutrally charged glycine (G). Increasingly hydrophobic peptides are created by substituting the neutral glycine (G) with alanine (A) and leucine (L). For this study, the peptide length was *N* = 48 unless otherwise stated.

The dense coacervate phase was then separated by centrifugation at 21475*g* at 4 °C for 20 min. The supernatant was moved to another tube, and only the sedimented complex coacervate phase was used in thermostability studies. For all the thermostability studies, the release of the virus from the complex coacervate phase was done by adding 50 μL of disassembly solution composed of 2 M sodium chloride followed by a 5 s vortex. The titer of the infectious virus in the supernatant and the coacervate phases was determined in triplicate using an MTT assay and reported with the standard deviation as the error. Studies to determine coacervate disassembly in different solutions were conducted using the same procedure, but with solutions composed of sodium chloride and sucrose with concentrations of each component ranging from 0 to 2 M. The partition coefficient for PPV is defined as the ratio of the titer in the coacervate divided by the ratio in the supernatant (eq 1). The log reduction value (LRV) is defined in eq 2

1 $$P=\frac{{\mathrm{titer}}_{c}}{{\mathrm{titer}}_{s}}$$

2 $$\mathrm{LRV}=\log\left(\frac{{\mathrm{titer}}_{t}}{{\mathrm{titer}}_{0}}\right)$$

where titer*_t_* is the remaining infectious viral titer at time *t* and titer_0_ is the initial viral titer, both in units of $\left(\frac{\mathrm{MT}T_{50}}{\mathrm{mL}}\right)$ .

The thermostability of encapsulated PPV was calculated by fitting the reduction in titer of the virus over time to a first-order kinetic model as shown in eq 3.

3 $$\ln\left(\frac{\mathrm{tite}r_{t}}{\mathrm{tite}r_{0}}\right)=-\mathrm{kt}$$

where *k* is the inactivation constant in $\left(\frac{1}{\mathrm{hour}}\right)$ and *t* is the time in (hours). Accordingly, the decay rate, the time required to reduce the population of infectious viruses by one log, can be calculated as

4 $$D=\frac{2.3026}{k}$$

### Animal Studies

All animal work was conducted in accordance with Institutional Animal Care and Use Committee guidelines at Texas Technological University Health Sciences Center IACUC (protocol number 23011). Ten BALB/c mice per group were each immunized twice (intramuscularly), at 2-week intervals, with 10^6^ MTT_50_ of PPV stored at 4 °C (V), PPV treated at 60 °C for 3 days (VT), coacervated PPV stored at 4 °C (CV), coacervated PPV treated at 60 °C for 3 days (CVT), or coacervate control with no PPV (C). Samples CV, CVT, and C were disassembled with 0.5 M sodium chloride and 1.0 M sucrose to a final concentration of 7 mM on an ionizable monomer basis. All volumes were matched so the final concentration of virus was the same in all samples. Then, all samples were mixed with alum hydroxide adjuvant prior to immunizations. Naïve mice were not immunized. Two months after the last immunizations, sera were collected from mice and anti-PPV IgG antibodies in sera were tested by ELISA (enzyme-linked immunosorbent assay) using purified PPV as the target antigen. Wells of ELISA plates were coated overnight at 4 °C with 10^6^ MTT_50_ of purified PPV. The wells were blocked with 0.5% nonfat milk for 2 h, and serial dilutions of sera from immunized mice, including sera from naïve mice, were added. The plates were incubated for 2 h and 1:5000 dilution of horseradish peroxidase-conjugated goat anti-mouse IgG antibodies (Jackson Immunoresearch, West Grove) was added for 1 h. The plates were developed by adding 3,3′,5,5′-tetramethylbenzidine, and the reaction was stopped by adding 1 M HCl. Serum reactivity at 1:2560 dilution was used to determine optical density at 450 nm (OD450).

### Statistical Analysis

A two-tailed unpaired *t* test assuming unequal variances was used to compare statistical differences between the data sets. A *p* value of less than 0.05 was considered significant. All data sets were analyzed in triplicate. The standard deviation for all of the data points was calculated in Microsoft Excel.

## Results and Discussion

The goal of this study was to determine the effect of polypeptide chain length and sequence on the thermal stability of porcine parvovirus (PPV) in a coacervate system. Previously, we had demonstrated that coacervates of poly­(lysine) and poly­(glutamate) with degree of polymerization *N* = 400 (*i.e*., K_400_, E_400_) could significantly improve the thermostability of PPV.^16^ We also determined that high levels of PPV incorporation could be achieved using coacervates made from shorter polypeptides (*N* = 48), where it was possible to explicitly define the sequence of amino acids using solid-phase synthesis.^32^ Here, we looked to determine how peptide sequence and chain length affect the thermal stability of PPV. We further wanted to determine whether coacervate stabilization of PPV would disassemble and reveal immunological epitopes and spur an immunological response *in vivo*.

PPV is a nonenveloped virus that forms an icosahedral capsid 18–26 nm in diameter with very regular T1 triangulation and an isoelectric point of 4.8–5.^54–56^ Given the net negative charge of the capsid at pH 8.0, and the importance of electrostatic interactions in driving coacervation, one might expect that the highest levels of PPV partitioning into the coacervate would occur at conditions where higher amounts of cationic poly­(lysine) would be present. This coacervate composition would thus allow for sufficient poly­(lysine) to complex with both anionic poly­(glutamate) and the virus. However, analysis of virus partitioning (P) as a function of polypeptide stoichiometry showed a range of outcomes, depending on the length and chemistry of the coacervating peptides (Figure 2).^32^ For example, the maximum partitioning of PPV shifted from the expected net-positive coacervate compositions observed with K_48_–E_48_ to a lower charge fraction with increasing polypeptide length. The addition of neutral or hydrophobic residues into the polycation, as shown in Figure 1b (forming a coacervate with E_48_), shifted the peak to higher cationic charge fractions, while the incorporation of neutral residues into the polyanion (coacervating with K_48_) allowed for reasonable virus incorporation at lower cationic charge fractions (Figure 2).

**Figure 2:**
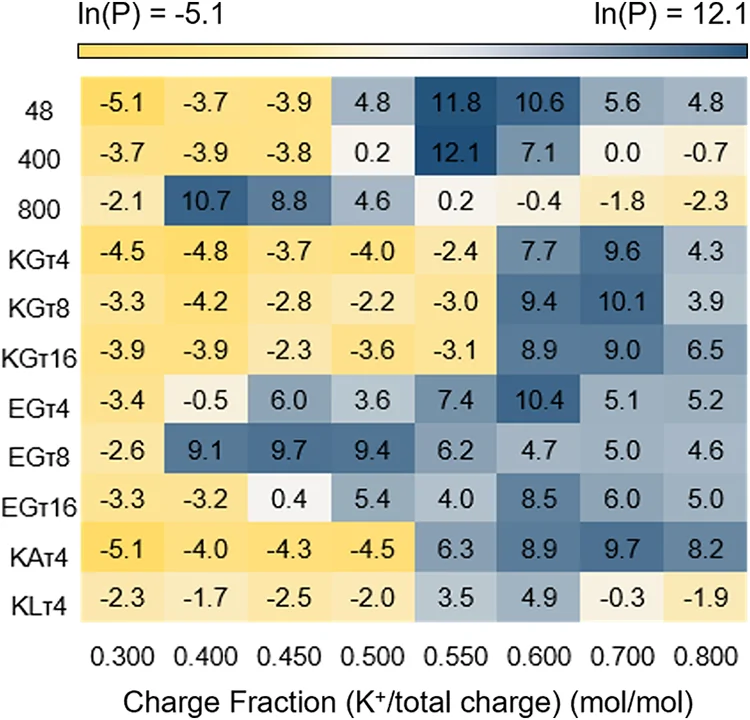
Heatmap depicting PPV partitioning in various coacervate systems at different charge fractions. For detailed analysis of the PPV behavior in the selected coacervate systems, refer to ref 32. Values are the average of three independent experimental replicates.

### Thermostability of Encapsulated Virus in Complex Coacervates

We selected the coacervate composition with the highest levels of PPV incorporation for subsequent thermostability studies (see Figure 2). Accelerated aging experiments to characterize the thermal stability of the various PPV-coacervate formulations were performed at 60 °C over the course of 7 days.^16^ Although various phase-separating peptide systems such as elastin-like polypeptides^57^ are known to be temperature sensitive, complex coacervates tend to be relatively temperature insensitive^58^ because of the dominant role of electrostatic interactions. Temperature-driven phase transitions have only been reported for a very small number of more hydrophobic synthetic polymer systems, and largely at extreme conditions of salt.^59,60^ Thus, we do not anticipate significant temperature effects on the phase behavior of the coacervates in our system. Additionally, our experiments were performed by removing the supernatant to isolate the coacervate prior to heating at 60 °C. With regard to correlating the results of our accelerated aging experiments to more realistic storage conditions, Simonelli and Dresback’s shelf life determination with a Q_10_ factor of 2 suggests that these experimental conditions should be similar to a 3-month study at 22 °C.^61,62^

We compared the titer loss of PPV encapsulated in our various coacervate environments with two control systems, where PPV was unencapsulated and suspended in PBS pH 7.2 or HEPES buffer pH 8.0. These buffers represent standard PPV conditions (PBS) and typical coacervate conditions (HEPES). For both control samples, the loss of viral titer (Figure 3a) and thus the maximum log reduction value (LRV) for the titer used were reached on the fifth day (Figure 3b). In contrast, and consistent with our previous report,^16^ the incorporation of PPV into a polypeptide-based coacervate resulted in enhanced thermal stability.

**Figure 3:**
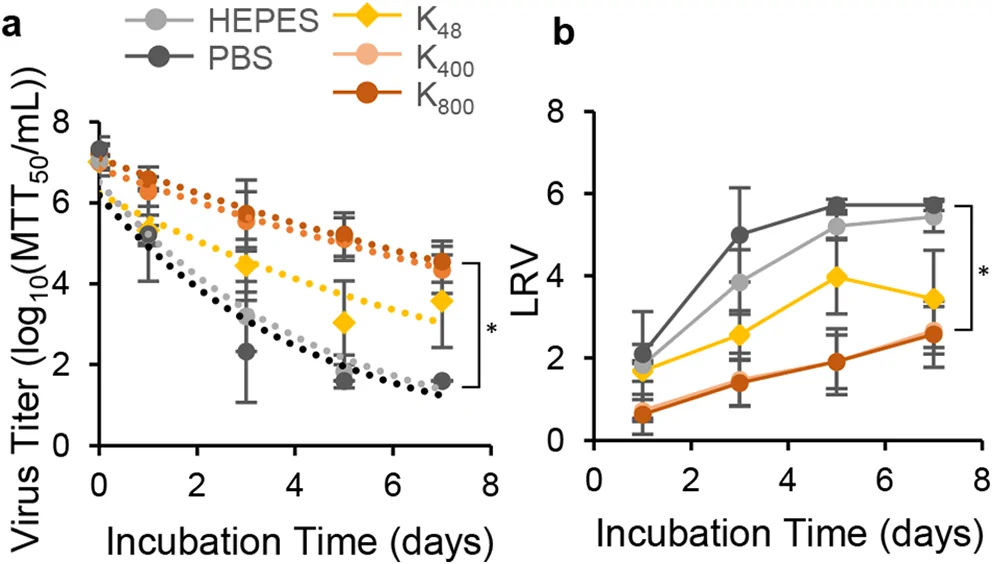
Thermal inactivation kinetics of PPV at 60 °C for (a) varying polypeptide length systems, also shown as (b) log reduction value (LRV). Two control systems, 10 mM HEPES pH 8.0 and 1× PBS pH 7.2, were used to compare the inactivation kinetics. Data presented are the average of three independent experimental replicates of inactivation, and the error bars are standard deviations. Dashed lines in (a) indicate curve fitting of eq 3. *p < 0.05 using the two-tailed *t* test.

### Thermostability and Polypeptide Chain Length

To begin with, we examined the effect of polypeptide length. The coacervate system with the shortest, 48-mer polypeptides (K_48_–E_48_) showed an increase in LRV of almost 2 logs within a day, representing an improved thermal stability from the PBS control, but not a statistical improvement compared with the HEPES control (Figure 3). However, the encapsulated virus LRV was significantly lower than the control systems at incubation times greater than 3 days. The maximum LRV for the titer used was reached on the fifth day for the control samples. The PPV in the 48-mer coacervate did not reach the limit of detection through the seventh day, which indicated a higher virus thermostability upon encapsulation (Figure 3). The longer polypeptides (K_400_–E_400_, K_800_–E_800_) were significantly better at thermally stabilizing the virus. We observed less than 1 LRV after 1 day and ∼2.5 LRV after 7 days, which translated to a slower virus decay rate, as shown in Figure S2a and Table 1.

**Table 1:** Thermal Inactivation Kinetic Constants and Decay Rates of PPV at 60°C and Varying Polypeptide Length^a^

system	inactivation constant (k) (day^–1^)	standard error of slope (SE_k_)	coefficient of determination (*R* ^2^)	decay rate (*D*) (day)
K_48_	1.46	0.23	0.91	1.58
K_400_	0.92	0.06	0.99	2.50
K_800_	0.93	0.05	0.99	2.47
(K_2_G_2_)_12_	1.41	0.17	0.97	1.64
(K_4_G_4_)_6_	1.50	0.16	0.98	1.54
(K_8_G_8_)_3_	1.26	0.15	0.96	1.83
(E_2_G_2_)_12_	1.68	0.23	0.96	1.37
(E_4_G_4_)_6_	1.27	0.15	0.97	1.81
(E_8_G_8_)_3_	0.77	0.07	0.97	3.00
(K_2_A_2_)_12_	0.91	0.05	0.99	2.52
(K_2_L_2_)_12_	1.45	0.15	0.98	1.58
(K_2_A_2_)_12_–(E_8_G_8_)_3_	0.67	0.12	0.90	3.42
HEPES	2.11	0.23	0.98	1.09
PBS	2.34	0.34	0.95	0.98

a Two control systems, 10 mM HEPES pH 8.0 and × PBS pH 7.2, were used to compare the inactivation kinetics. Inactivation constants and decay rates were calculated using eqs 3 and 4. The decay rate over time can be found in Figure S2.

While we observed a decrease in the decay rate from 48-mer to 400-mer, there was no significant difference in the decay rate upon increasing the chain length from 400-mer to 800-mer (Figure S2a). We hypothesize that the length-dependent improvements in stabilization could be a consequence of enhanced crowding due to increases in the polypeptide concentration in the coacervate phase.^63^ Furthermore, this hypothesis is consistent with the attenuation of effects seen at the longer chain lengths, as aspects of coacervate phase behavior have been previously shown to attenuate above *N* ∼200.^63–66^ However, we would note that there remains the possibility for composition-dependent effects, as maximum PPV incorporation occurred at a higher poly­(lysine) fraction for the shorter polypeptides compared to the longer ones (Figure 2), meaning that the stabilization experiments were performed at different polypeptide compositions. Atomistic simulations or direct measurement of the interacting species within the coacervate would be needed to determine the potential impact of such compositional effects and are beyond the scope of the current work.

### Thermostability and Polypeptide Charge Patterning

While longer polypeptides facilitated a more thermostabilizing environment, solid-phase peptide synthesis of sequence-controlled polypeptides was limited to shorter chain lengths. We synthesized a library of 48-mer different lysine-*co*-glycine (KG) and glutamate-*co*-glycine (EG) polypeptides with regular repeating blocks of the two types of amino acids. Each of the peptides is half-charged, meaning that 24 of the 48 residues are glycine, and the size of the repeating block is described by the parameter τ (Figure 1b). For example, a KG peptide with τ = 4 consists of a repeating unit of 4 amino acids, two lysine and two glycine (i.e., (K_2_G_2_)_12_). Different-sized blocks (τ = 4,8,16) were used to determine the impact of the size of the block, with coacervates formed using one patterned polypeptide and a homopolypeptide (e.g., (K_2_G_2_)_12_–E_48_ or K_48_–(E_2_G_2_)_12_). The use of a half-charged patterned polypeptide is expected to decrease the concentration of polypeptide in the coacervate when compared with the K_48_–E_48_ system.^46,47,63^ However, the concentration and correspondingly the viscosity of the coacervate has been shown to increase with increasing charge block size (τ).^45^

Coacervates formed from KG-based peptides showed a slight shift in the maximum partitioning condition to higher cationic charge fractions, as compared with the K_48_–E_48_ system (Figure 2). However, as shown in Figure 4a, coacervation with these charge-patterned polycations did not result in a statistical improvement in the thermal stability of PPV compared to the fully charged 48-mer. EG-based coacervates similarly showed only minor differences in the condition for maximum uptake, with the exception of the τ = 8, K_48_–(E_4_G_4_)_6_ system, which shifted to a lower cationic charge fraction (Figure 2). Here again, the smaller block sizes (τ = 4,8) did not show improved stability compared to the fully charged 48-mer system (Figure 4b). However, the τ = 16 system of K_48_–(E_8_G_8_)_3_ showed higher stability than even the previously best-performing 800-mer system.

**Figure 4:**
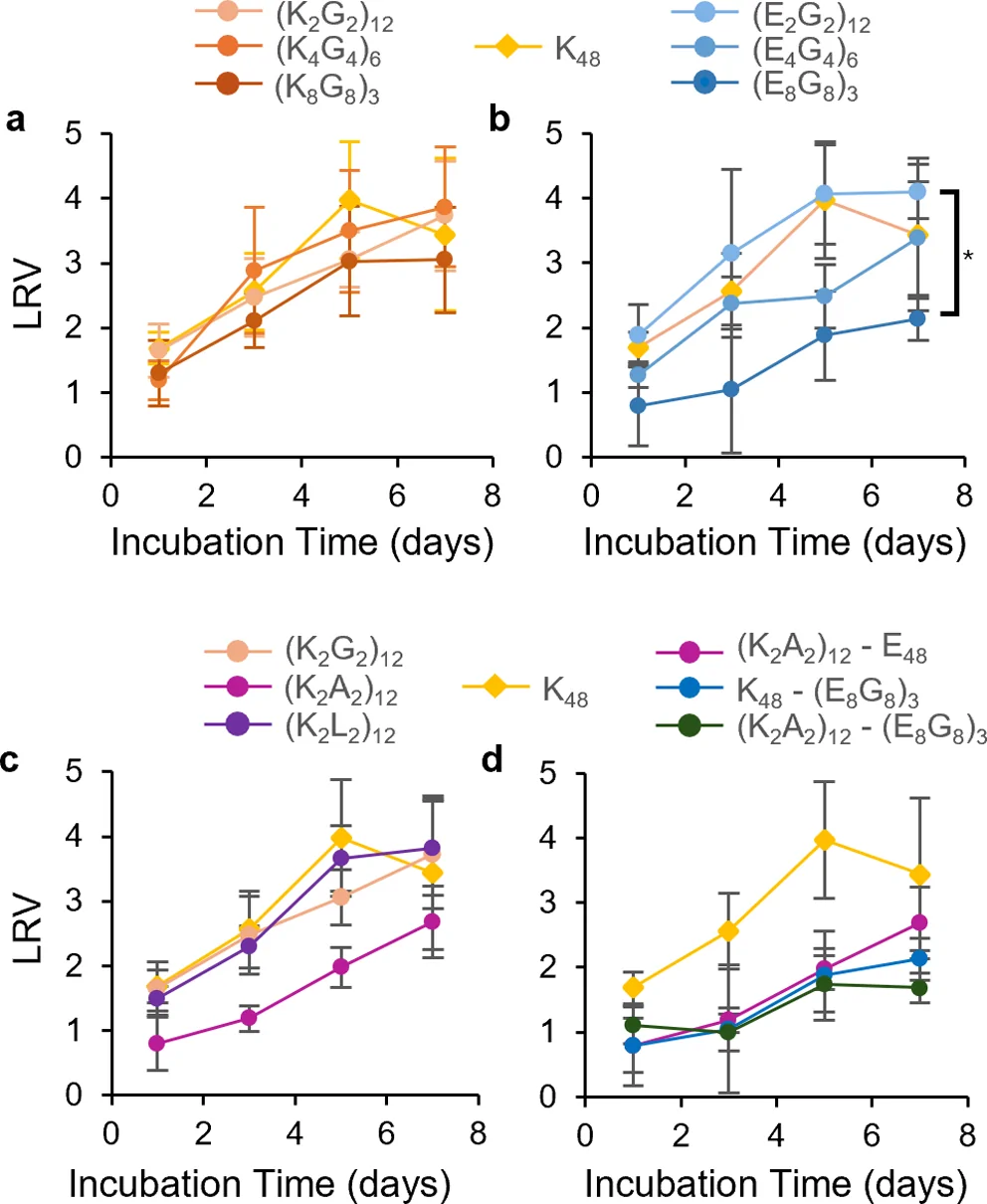
Thermal inactivation of PPV at 60 °C in block-*co*-polypeptides with neutral glycine blocks: (a) block-*co*-poly­(KG) with fully charged E_48_ and (b) block-*co*-poly­(EG) with fully charged K_48_. (c) Thermal inactivation of PPV in coacervates containing hydrophobic residues with fully charged E_48_. (d) Thermal inactivation comparison among single-patterned polypeptide and double-patterned polypeptides. (K_2_A_2_)_12_ (τ = 4) and (E_8_G_8_)_3_ (τ = 16) were used as the patterned polypeptides as the systems were the most successful in stabilizing PPV of the polypeptide system tested. Data presented are the average of three independent experimental replicates of inactivation, and the error bars are standard deviations. The instantaneous titers over the course of 7 days can be found in Figure S3. *p < 0.05 using the two-tailed *t* test.

The performance of the K_48_–(E_8_G_8_)_3_ system indicates a trend of a decreasing LRV as a function of block size for the EG-series peptides. Although it is possible that a similar but weaker trend exists for the KG-series, the uncertainties associated with the log-dilution nature of the viral titer assay prevent describing this trend with statistical certainty. In the context of only these half-charged peptides, this trend of improved stability with increased block size is consistent with our previous observation of increased thermostabilization with longer-chain coacervates that have higher concentrations of polypeptide, or relatedly a higher viscosity (Figure 5).^45^ However, such physical crowding arguments cannot explain the improved stability of the K_48_–(E_8_G_8_)_3_ system relative to the K_48_–E_48_ system, let alone that of K_800_–E_800_. Thus, some aspect of molecular-level interactions between the virus and the coacervate must be responsible for improved stability.

**Figure 5:**
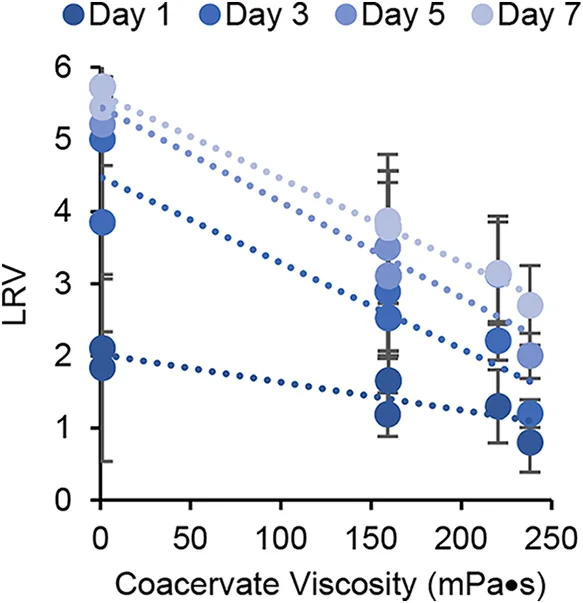
Thermal inactivation of PPV at 60 °C as a function of sample viscosity and time. The viscosity for the HEPES and PBS buffer systems was estimated as 1 mPa•s. Viscosity data for the KG-series and KA τ = 4 system were adapted from.^45^ Data presented are the average of three independent experimental replicates of inactivation, and the error bars are standard deviations. The instantaneous titers over the course of 7 days can be found in Figure S3.

In the absence of knowledge regarding specific binding motifs present on the surface of the net negatively charged PPV capsid, the KG-series coacervates would be expected to experience the strongest interactions with the largest τ = 16 KG block size. In contrast, the decrease in charge of the EG-series coacervates would be expected to result in stronger interactions between the capsid and K_48_ that are maximized in the presence of the smallest τ = 4 EG system. However, our results suggest that optimal stabilization occurs as a consequence of physical crowding, paired with an optimal set of intermolecular interactions that are neither too weak nor too strong. Additional studies should be conducted using other viruses and with additional polypeptides and/or copolymers to assess if these trends extend to other systems, and how the chemistry of the virus capsid might affect thermostabilization. Such efforts would also benefit from the ability to directly visualize and quantify such interactions via atomistic simulations, though we acknowledge the computational challenge of modeling such large systems.^67^

### Thermostability and Polypeptide Hydrophobicity

Glycine was used in the initial patterned polypeptides as a relatively simple, neutral, yet hydrophilic residue. Moreover, glycine is widely used in biotherapeutic formulations due to its ability to stabilize proteins against various environmental stresses.^68–70^ However, we can also take advantage of the chemistry of the neutral residues to examine the effect of hydrophobicity on PPV incorporation and thermostabilization. To this end, we leveraged the τ = 4 series of lysine-based polypeptides to limit the potential for solubility issues and changed the neutral residue from glycine (G) to alanine (A) and leucine (L), as shown in Figure 1b. We chose to explore the effect of hydrophobicity only for the polycation as we looked to enhance its interaction with the negatively charged capsid. Furthermore, PPV is known to be more hydrophobic than many proteins,^71^ and so could potentially benefit from enhanced hydrophobic interactions.

As was the case for the KG-based coacervates, only minor shifts in the conditions for maximum partitioning were observed when the neutral residue was changed to alanine (KA) or leucine (KL), and there was not a correlation between PPV incorporation and thermal stability. For these types of systems, the concentration of polypeptide in the coacervate and viscosity have been shown to increase with hydrophobicity.^45^ Interestingly, we observed that the KA-based coacervate resulted in better PPV thermostability compared to the KG- and KL-systems, which performed similarly to K_48_–E_48_ (Figure 4c). Furthermore, the stability of the KA-based system was similar to that of the 800-mer system.

The data in Figure 5 indicate that the performance of the (K_2_A_2_)_12_–E_48_ is consistent with the previously discussed trend of increasing stabilization with increasing peptide viscosity. While data were not available in the literature for the specific τ = 4 KL-system used in this study, it is reasonable to expect that it should have a higher viscosity than that of the KA system,^45^ suggesting a nonmonotonic shift in the data. Here again, our results suggest the presence of optimal conditions that take advantage of a balance of physical crowding, paired with an optimal set of intermolecular interactions. While the τ = 4 KG-system might have lacked sufficient crowding to stabilize the virus, it is possible that the hydrophobicity of the KL-system resulted in coacervates that are too strongly self-associating because of their hydrophobicity. This possibility of strong associations is consistent with the observed decreases in PPV partitioning into the KL-coacervates (Figure 2), though more direct measures of binding would be needed to confirm this hypothesis.

Having identified both a polycation and a polyanion that individually showed improvement in PPV stability, we looked to combine the τ = 4 (K_2_A_2_)_12_ with the τ = 16 (E_8_G_8_)_3_ to test for synergistic performance. While the maximum partition conditions for the two individual systems were slightly different, we selected a cationic charge fraction of 0.6 for subsequent incorporation and thermostability tests. The incorporation of virus into the coacervate was significantly decreased when both the polycation and polyanion were patterned (Figure S4), consistent with previous data on proteins.^72^ Despite the lower levels of virus incorporation, the LRV was slightly lower for the combined (K_2_A_2_)_12_–(E_8_G_8_)_3_ system on day 7, compared to the parent (K_2_A_2_)_12_–E_48_ or K_48_–(E_8_G_8_)_3_ systems (Figure 4d), though not statistically significant.

### Effect of Sugar Excipients on the Thermostability of Encapsulated Virus

To explore the combined potential of coacervation and small-molecule excipients, we tested two disaccharides, sucrose and trehalose, as additives in the fully charged K_48_–E_48_ system. In the absence of virus, we had previously determined that neither sucrose nor trehalose partitions preferentially into the dense coacervate phase (i.e., partition coefficient of approximately 0.30 for sucrose and 0.03 for trehalose).^73^ Thus, for the 0.5 and 0.8 M concentrations tested here, assuming that the presence of virus does not alter the excipient partitioning, we would expect a concentration of approximately 0.15 and 0.24 M sucrose in the coacervate and 0.015 and 0.024 M for trehalose. While trehalose did not significantly improve the thermostability of PPV in the coacervate phase, likely because trehalose has a low concentration in the coacervate phase, we did observe an improvement in stability with sucrose. (Figure 6). The stabilizing effect of sucrose has been shown on other nonenveloped viruses, including adenovirus^74,75^ and human rotavirus,^76^ as well as on enveloped viruses such as influenza virus.^7^ That sucrose was able to improve the stability of PPV even in the dense context of a polypeptide-based coacervate is noteworthy and suggests that further stabilization might be achieved through a survey of more diverse polypeptide chemistries and/or the inclusion of more diverse small-molecule excipients. Additionally, the extension of this approach to the stabilization of enveloped viruses would be an interesting area for future work, particularly given our previous report, which suggested the potential for increased recovery of two herpes viruses in the coacervate phase when trehalose was present.^73^

**Figure 6:**
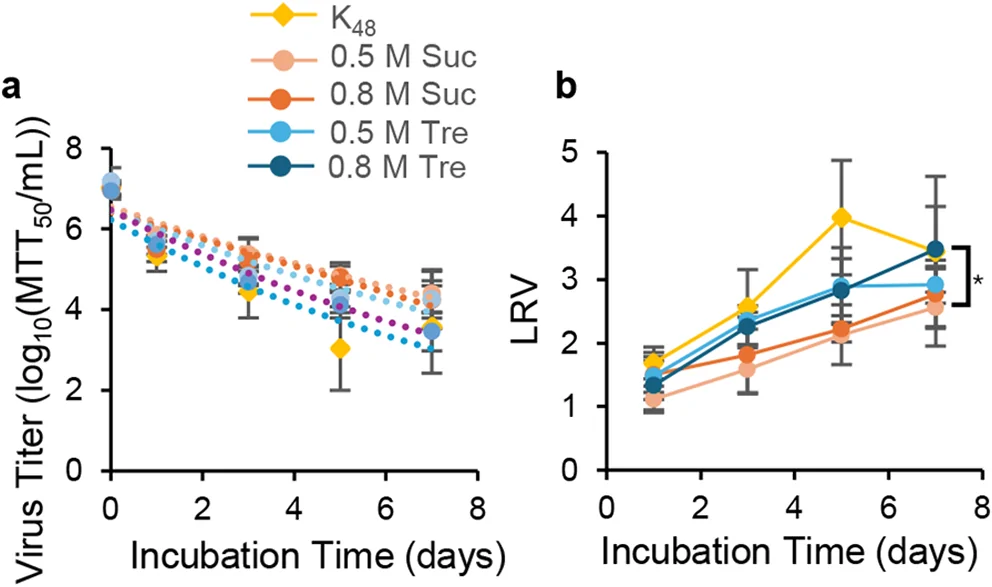
Thermal inactivation of PPV at 60 °C in 48-mer coacervate system containing disaccharides. (a) The instantaneous virus titers and (b) LRV over the course of 7 days. Sucrose and trehalose were used as the disaccharide additives at 0.5 and 0.8 M concentration in the total system. Data presented are the average of three independent experimental replicates of inactivation, and the error bars are standard deviations. Dashed lines indicate curve fitting of eq 2. *p < 0.05 using the two-tailed *t* test.

### *In Vivo* Immune Response

Lastly, we explored the potential for the coacervate-stabilized virus to produce an immune response *in vivo* using the 400-mer coacervate system. We had previously demonstrated that our coacervate materials showed very minimal cytotoxicity in PK-13 cells,^16^ consistent with other reports.^20^ In planning this experiment, we decided to first disassemble the coacervate to present the virus sample at a similar concentration for all samples. While coacervate disassembly for viral titer experiments had involved the use of 2 M NaCl, this high-salt approach could not be used for injectable samples since high NaCl concentrations can induce muscle pain and other adverse effects after intramuscular injection.^77,78^ Previous work had demonstrated that mixtures of sucrose and NaCl could be used to disassemble coacervates.^73^ Therefore, we evaluated the potential for using mixtures of sucrose and lower NaCl concentrations to release PPV, using viral titer as an indicator.

The disassembly of the coacervate in 2 M NaCl was used as the control for disassembly. When disassembly was attempted using varying concentrations of sucrose without NaCl (Figure 7), a lower virus titer than the 7.1 log_10_(MTT_50_/mL) for the 2 M NaCl control was observed, consistent with our previous results that disassembly had not been achieved.^73^ However, the use of 0.5 M NaCl in tandem with varying concentrations of sucrose was able to recapitulate the titer of 2 M NaCl. We also explored the toxicity of the disassembled coacervates (Figure S5) on Vero cells. We chose Vero cells because they are primate cells and thus closer to human injection than are porcine cells. PK-13 (our porcine cell line that grows PPV) is also known to be extremely hardy, and thus, the more sensitive Vero cell line was used. The disassembled coacervates were not any more toxic than the disassembly solutions. Ultimately, we used a mixture of 1 M sucrose and 0.5 M NaCl for coacervate disassembly, as this result performed similarly to the baseline 2 M NaCl sample but minimized the overall concentrations of both salt and sucrose.

**Figure 7:**
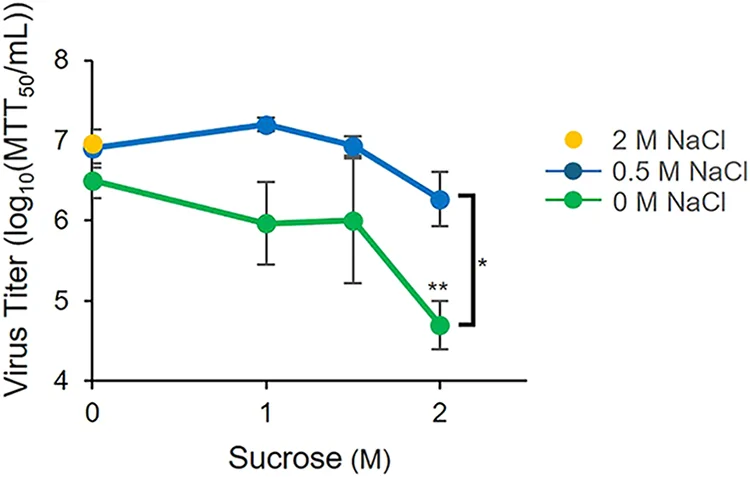
PPV titers of coacervates composed of PPV and K_400_–E_400_ after disassembly with buffers composed of different concentrations of sucrose and sodium chloride (NaCl). Data presented are the average of three independent experimental replicates, and the error bars are standard deviations. *p < 0.05 using the two-tailed *t* test. **p < 0.05 as compared to the 2 M NaCl control using the two-tailed *t* test.

We measured the immunogenicity of our various PPV formulations with the K_400_–E_400_ system via the production of PPV-binding antibodies following a vaccine-like treatment regimen that included an alum adjuvant. As expected, the virus control (V) that had been stored at 4 °C and maintained infectivity gave the highest immunogenicity response (Figure 8 and end point dilution titer in Figure S6). PPV after 3 days at 60 °C (VT) showed a significant, though lower immune response, even though the sample no longer had measurable infectivity. Surprisingly, although the coacervate-based PPV sample (CV) showed higher immunogenicity than the coacervate sample that was subjected to 3 days at 60 °C (CVT), the response for the coacervate-based PPV samples was significantly lower than for the virus alone. These data indicate PPV infectivity may not correlate with immunogenicity. However, it is also possible that the lack of salt and sugar in the V and VT samples, due to the high dilution it would have caused, as compared to the coacervated samples, could have also interfered with the action of the alum adjuvant.

**Figure 8:**
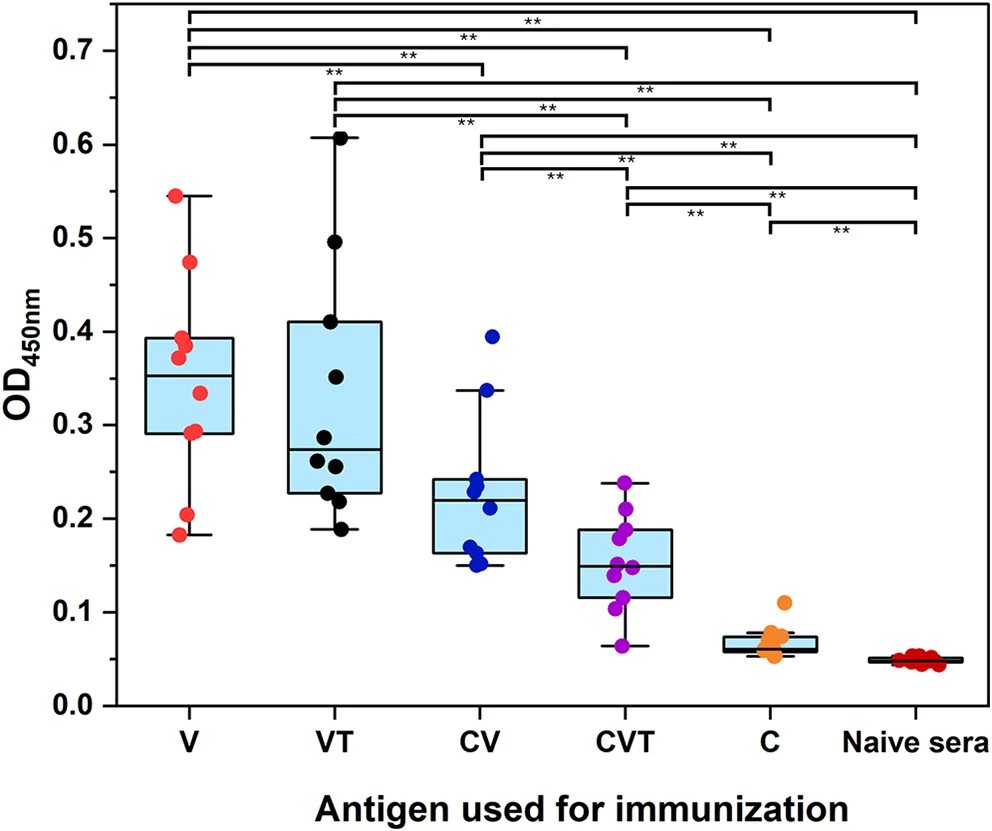
Immunogenicity of PPV in mice. Mice were immunized twice with the antigens in the presence of alum hydroxide adjuvant. Sera were collected from mice and were used to determine IgG levels by ELISA. Each datum represents OD450 in each mouse at 1:2560 dilution. V: virus only stored at 4 °C, VT: virus samples after 60 °C for 3 days, CV: virus samples in coacervates stored at 4 °C, CVT: virus samples in coacervates after 60 °C for 3 days, C: coacervate only without virus. Statistical analysis used the two-tailed unpaired *t* test. **p < 0.05.

Our initial assumption had been that the recovery of the infectivity from the coacervate (Figure 7) was proof that the virus was released from the coacervate phase. Direct inspection of the coacervate formulation under conditions of 0.5 M NaCl and 1.0 M sucrose confirmed that the coacervate had not been fully disassembled (see Figures S7 and S8). However, previous studies of protein uptake into coacervates had demonstrated that increases in ionic strength would dramatically reduce the partitioning of protein into the coacervate at levels far below the amount of salt needed to abrogate phase separation.^72^ Furthermore, the addition of the alum adjuvant (*i.e*., aluminum trihydroxide, a multivalent salt) would be expected to further enhance the release.^79^

The disparate performance from infectivity and immunogenicity would seem paradoxical. How is it that the molecular interactions associated with infectivity could avoid interference from the coacervating peptides, while those associated with generating an immune response are suppressed? One possible explanation is that the PPV was not, in fact, sufficiently released from the complexing peptides and that the strongest binding motifs associated with this interaction involve the epitopes associated with generating an immune response. A recent study by Adu et al. showed that the immune response following canine parvovirus infection in dogs generated only 2–3 dominant antibodies.^80^ Therefore, only two to three epitopes on the capsid are involved in generating a measurable immune response. However, the same antibodies did not completely neutralize virus infection when tested *in vitro*. These results indicate that regions of the capsid responsible for generating an immune response by the host are different from the ones involved in binding cell receptors, leading to infection, and could potentially explain our results. Potential strategies to address this challenge could include the use of shorter polypeptides and the use of sequence to both make coacervate disassembly easier at lower concentrations of salt/sugar and potentially avoid interactions with the relevant epitopes.

Another intriguing possibility is that the coacervating polypeptides adversely affect the interactions between PPV and the alum adjuvant. Previous studies have reported that enhanced binding of antigens to alum is associated with increased immune responses.^81^ The highly charged polypeptides present in the sample would be expected to compete with PPV for such binding sites, ultimately reducing the number and potentially the bioavailability of highly immunogenic antigens. Future studies should explore the effect of coacervating polypeptides on the immunogenicity of antigens formulated with alum.

## Conclusions

Improved thermostability could greatly reduce the cost of distributing vaccines, although the development of stabilizing formulations remains a significant challenge. Building upon previous work that polypeptide-based coacervates could provide thermal stability to PPV and that peptide sequence could modulate encapsulation efficiency,^16,32^ here, we explored how polypeptide sequence and length could affect the thermal stability of PPV in accelerated aging experiments. We observed that stability tended to increase with the polypeptide length for coacervates of unpatterned poly­(lysine) and poly­(glutamate). However, the use of charge patterning and the incorporation of different neutral amino acids allowed for improved stability with the shorter peptides required for solid-phase peptide synthesis and performance that equaled and even exceeded those of the longer unpatterned polypeptides. Our data suggest that PPV stabilization was achieved via a combination of both entropic crowding effects related to the concentration of polypeptide in the coacervate and enthalpic interactions and that in both instances an optimum condition existed that avoided the extremes of both crowding and interactions.

We also quantified the potential for combining coacervate stabilization of PPV with the addition of small-molecule excipients. Sucrose was able to enhance the thermostability of coacervate-encapsulated PPV, suggesting the potential for synergistic formulation strategies. We also leveraged a combination of sucrose and NaCl to facilitate the dissolution of PPV-containing coacervates for immune studies in mice. We found that both heat inactivation and coacervation lowered the immune response to PPV as measured by the production of PPV-binding antibodies. These results indicate that viral infectivity, which had been used as a measure of thermostability, does not necessarily corroborate with antibody production. Further investigations are needed to elucidate whether the apparent disconnect between infectivity and immunogenicity is a consequence of interactions between polypeptides and specific motifs on the capsid surface or interference between the coacervate matrix and the alum adjuvant. Though far beyond the scope of this work, understanding how biomolecular condensates (i.e., coacervate-like materials) modulate viral infectivity, replication, and the resulting host immune response could be another interesting area of study.

Overall, this work highlights the exciting ways that sequence and length effects in polypeptide-based complex coacervation can be leveraged for the incorporation and thermal stabilization of PPV and other viruses, proteins, and biomolecules. Ultimately, we aim to expand this work to include a diverse array of protein and viral systems to understand the molecular mechanisms by which the material microenvironment of a formulation can be tuned to create thermally stable therapeutics.

## Supplementary Material



## Notes

Table of counterions, molecular weights, and polydispersity index (PDI) of polypeptides (Table S1); MALDI-TOF analysis of polypeptides (Figure S1a–j); decay rate for the titer loss of PPV in different coacervate systems (Figure S2); PPV titers post-incubation at 60 °C in the systems at various block sizes in polycation or polyanion (Figure S3); PPV partitioning comparison in the systems with at least one homopolypeptide vs both block-*co*-polypeptides (Figure S4); toxicity of coacervates (Figure S5); end point dilution of *in vivo* immunogenicity (Figure S6); evaluation of coacervate disassembly with NaCl for the K400–E400 system (Figure S7); and evaluation of coacervate disassembly with 500 mM NaCl and sucrose for the K_400_–E_400_ system (Figure S8) (PDF)
