# Supplementary material for: Polypeptide Sequence Effects on Virus Thermostability in Complex Coacervate Formulations
Source: Biomacromolecules. 2026 Aug 7;27(9):5997–6008. doi: 10.1021/acs.biomac.6c00747 (PMC13580023; doi:10.1021/acs.biomac.6c00747)
Supplement: Supplementary file 1 [file bm6c00747_si_001.pdf]

Supplementary Information

Polypeptide Sequence Effects on Virus Thermostability in Complex Coacervate Formulations

Pratik U. Joshi[1,2], Claire Decker[1], Arvind Sathyavageeswaran[3, 4], Xianci Zeng[3,4], Hong Liu[5], Milad Kheirvari[5], Ebenezer Tumban[5], Lynn Manchester[1], Idris Tohidian[1,2], Eduardo Barbieri[2], Sarah L. Perry[3,4,*], Caryn L. Heldt[1,2,*]

[1]Department of Chemical Engineering, Michigan Technological University, USA
[2]Health Research Institute, Michigan Technological University, USA
[3]Department of Chemical and Biomolecular Engineering, University of Massachusetts Amherst, USA
[4]Institute for Applied Life Sciences, University of Massachusetts Amherst, USA
[5]Graduate Program in One Health Sciences, School of Veterinary Medicine, Texas Tech University, Amarillo, TX 79106, USA

*Corresponding authors: heldt@mtu.edu & perrys@engin.umass.edu

Raw Data found at: https://digitalcommons.mtu.edu/all-datasets/71

Table S1: Table of counter-ions, molecular weights and polydispersity index (PDI) of polypeptides.

Name	Counter Ion	M_w (g/mol)	M_n (g/mol)	N	PDI[e]
K_{48}	TFA^-	-	6,218[a]	48[a]	-
E_{48}	Na^+	-	6,236[a]	48[a]	-
K_{400}	TFA^-	97,000	95,300[b]	394[b]	1.08
E_{400}	Na^+	60,000	59,000[c]	391[c]	1.01
K_{800}	TFA^-	194,000	183,700[c]	759[c]	1.06
E_{800}	Na^+	120,000	118,400[d]	784[d]	1.06
$(K_2G_2)_{12}$	TFA^-	-	4,459[a]	48[a]	-
$(K_4G_4)_6$	TFA^-	-	4,465[a]	48[a]	-
$(K_8G_8)_3$	TFA^-	-	4,464[a]	48[a]	-
$(K_2A_2)_{12}$	TFA^-	-	4,800[a]	48[a]	-
$(K_2L_2)_{12}$	TFA^-	-	5,798[a]	48[a]	-
$(E_2G_2)_{12}$	Na^+	-	4,497[a]	48[a]	-
$(E_4G_4)_6$	Na^+	-	4,498[a]	48[a]	-
$(E_8G_8)_3$	Na^+	-	4,508[a]	48[a]	-

[a] Determined by MALDI-TOF. The weights of the TFA^- counterion are excluded from the reported M_n values

[b] Determined by light scattering, as reported by the manufacturer.

[c] Determined by ^1H NMR, as reported by the manufacturer.

[d] Determined by viscosity, as reported by the manufacturer.

[e] Determined by gel permeation chromatography (GPC), as reported by the manufacturer.

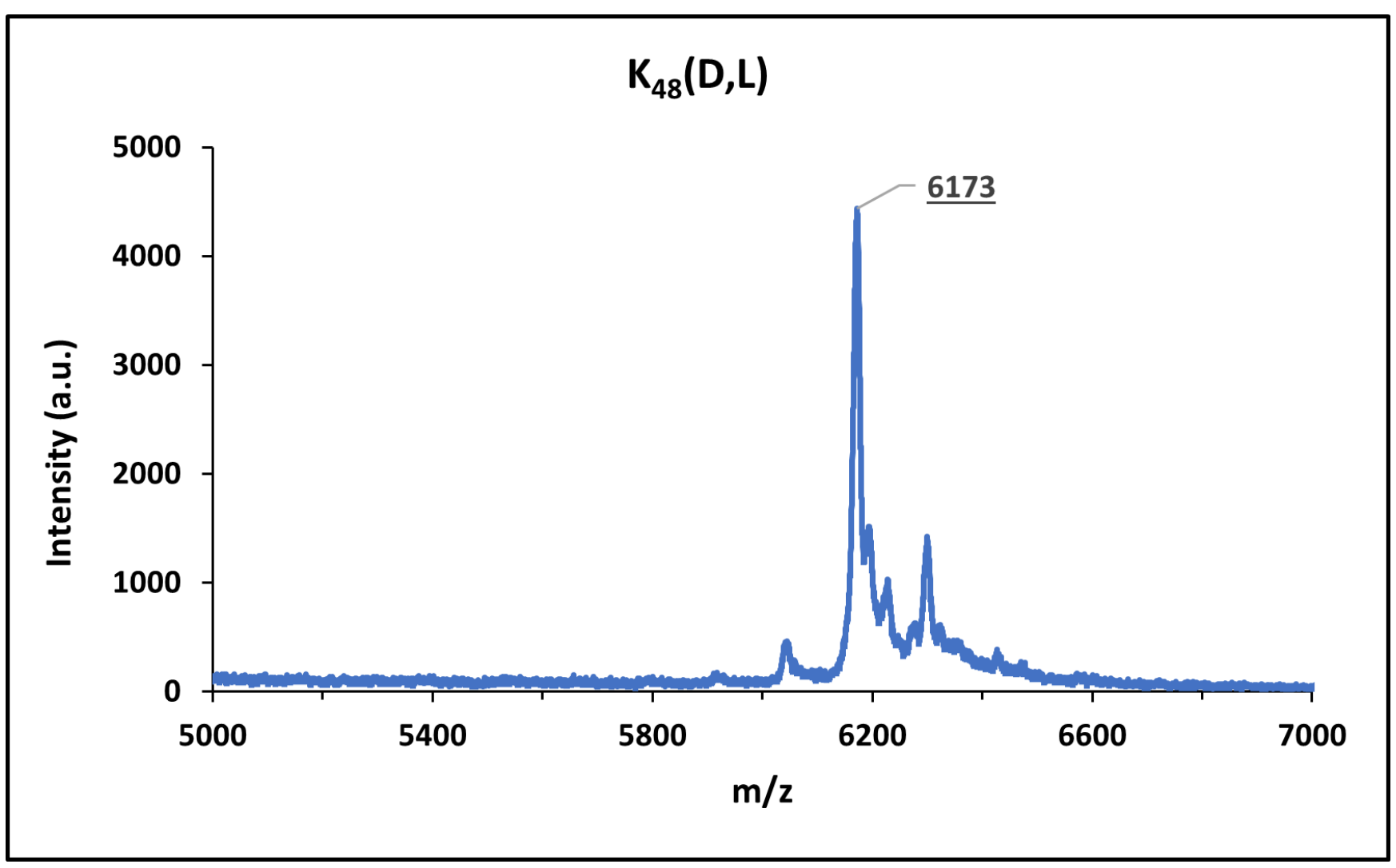

Figure S1a: MALDI-TOF analysis of (poly(*D,L*-lysine)), K_{48}. The expected theoretical mass is 6169.3 g/mol, excluding counterions. The maximum observed at m/z = 6173 is very close to the expected theoretical mass.

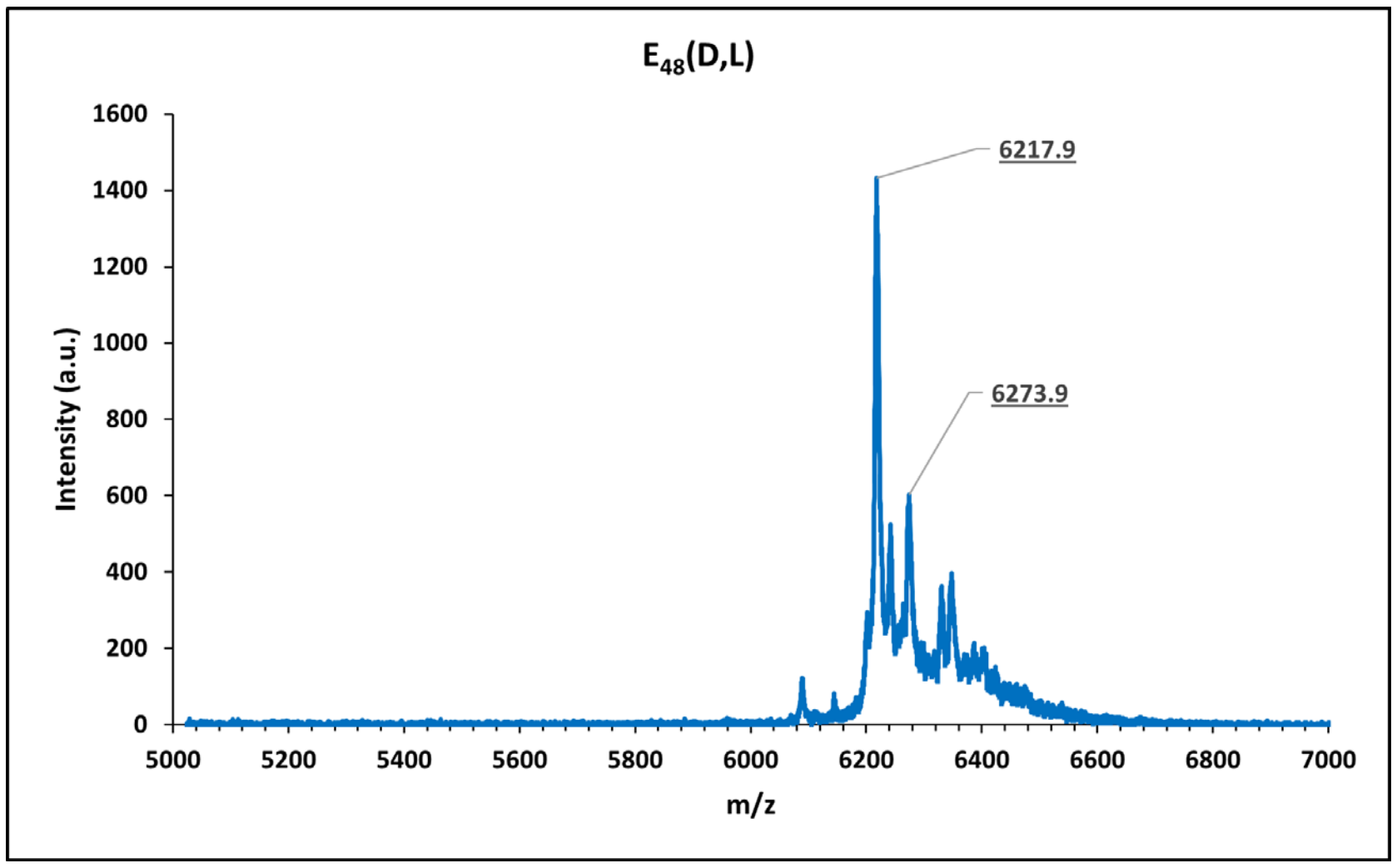

Figure S1b: MALDI-TOF analysis of poly(*D,L*-glutamate), E_{48}. The expected theoretical mass is 6214.5 g/mol, excluding counterions. The maximum observed at m/z = 6217.9 is very close to the expected theoretical mass. The peak at m/z = 6273.9 could correspond to the presence of a Na^+ and K^+ counterion.

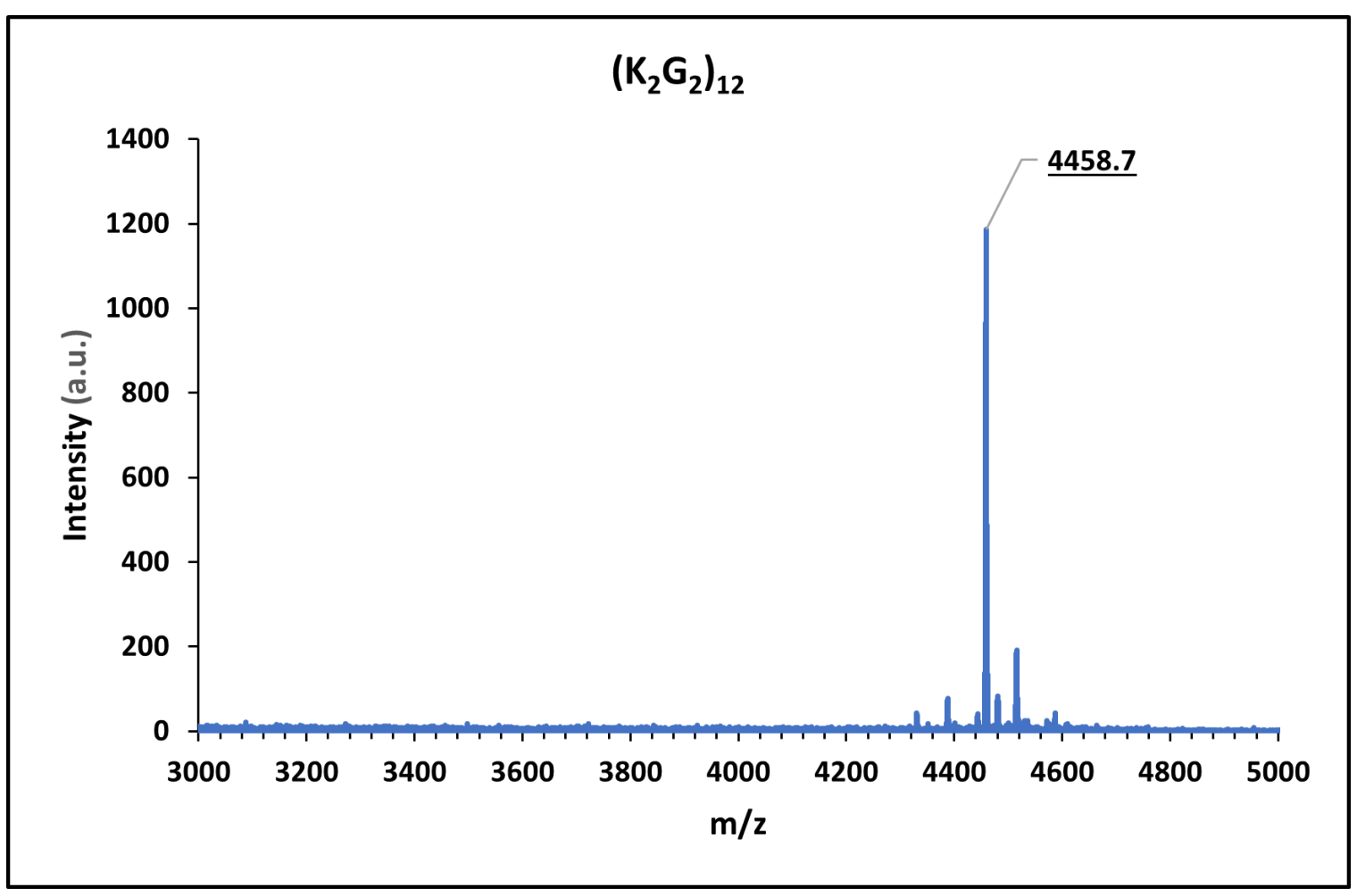

Figure S1c: MALDI-TOF analysis of poly(lysine-co-glycine), $(K_2G_2)_{12}$. The expected theoretical mass is 4462.4 g/mol, excluding counterions. The peak observed at m/z = 4458.7 is very close to the expected theoretical mass.

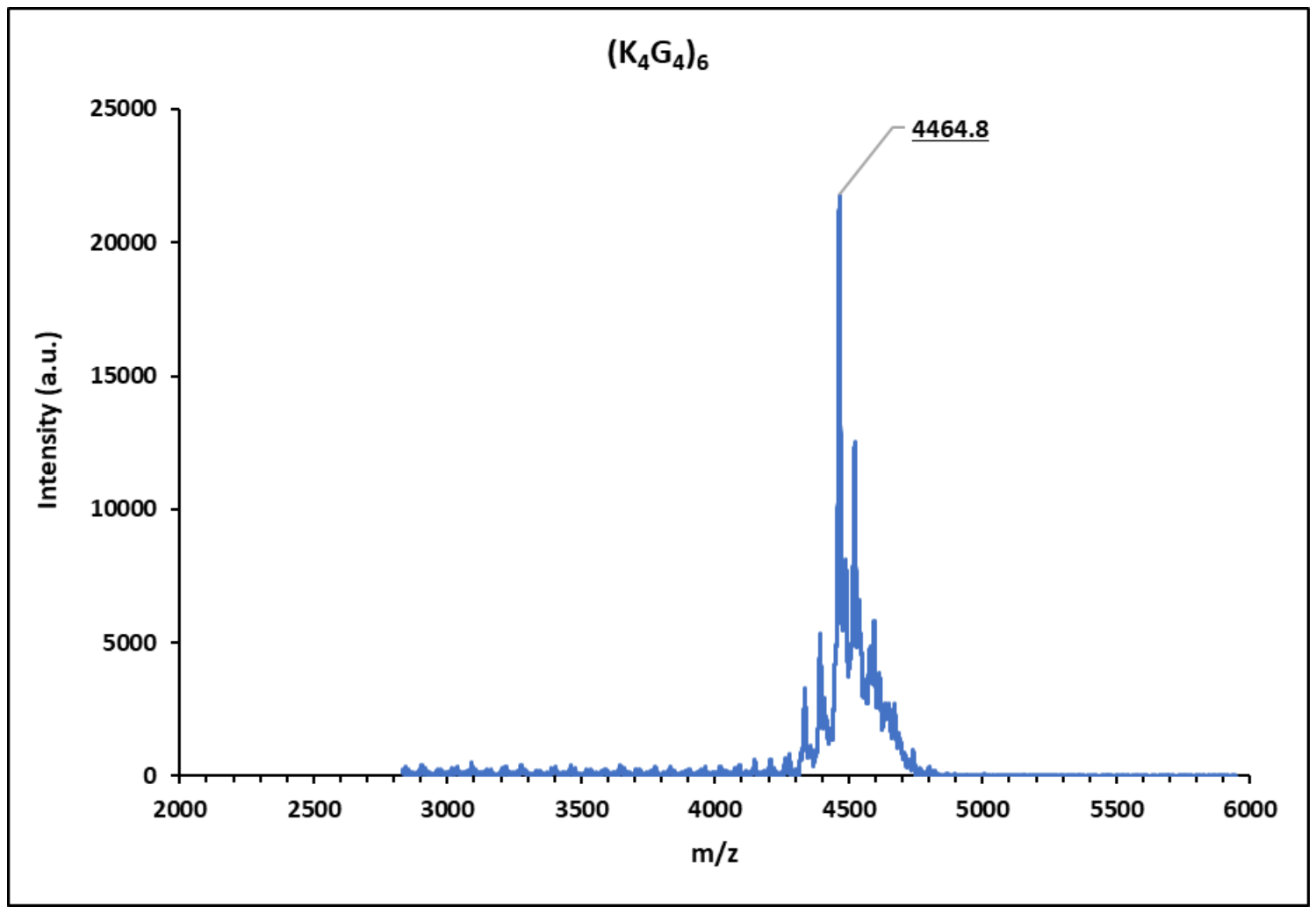

Figure S1d: MALDI-TOF analysis of poly(lysine-co-glycine), $(K_4G_4)_6$. The expected theoretical mass is 4462.4 g/mol, excluding counterions. The peak observed at m/z = 4464.8 is very close to the expected theoretical mass.

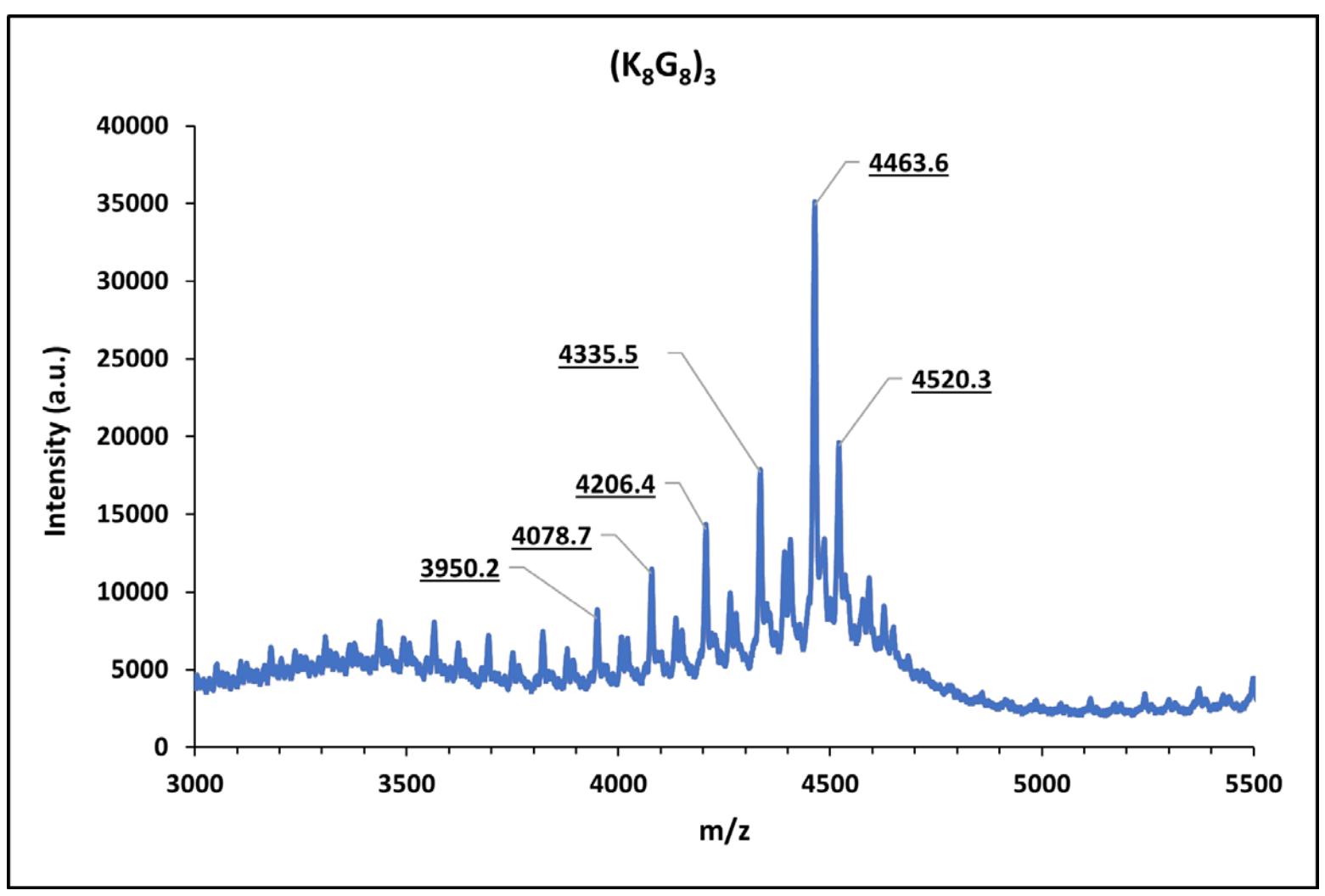

Figure S1e: MALDI-TOF analysis of poly(lysine-co-glycine), $(K_8G_8)_3$. The expected theoretical mass is 4462.4 g/mol, excluding counterions. The peak observed at m/z = 4463.6 is very close to the expected theoretical mass. The peaks at m/z = 4335.5, 4206.4, 4078.7, and 3950.2 suggest possible missing lysine residues.

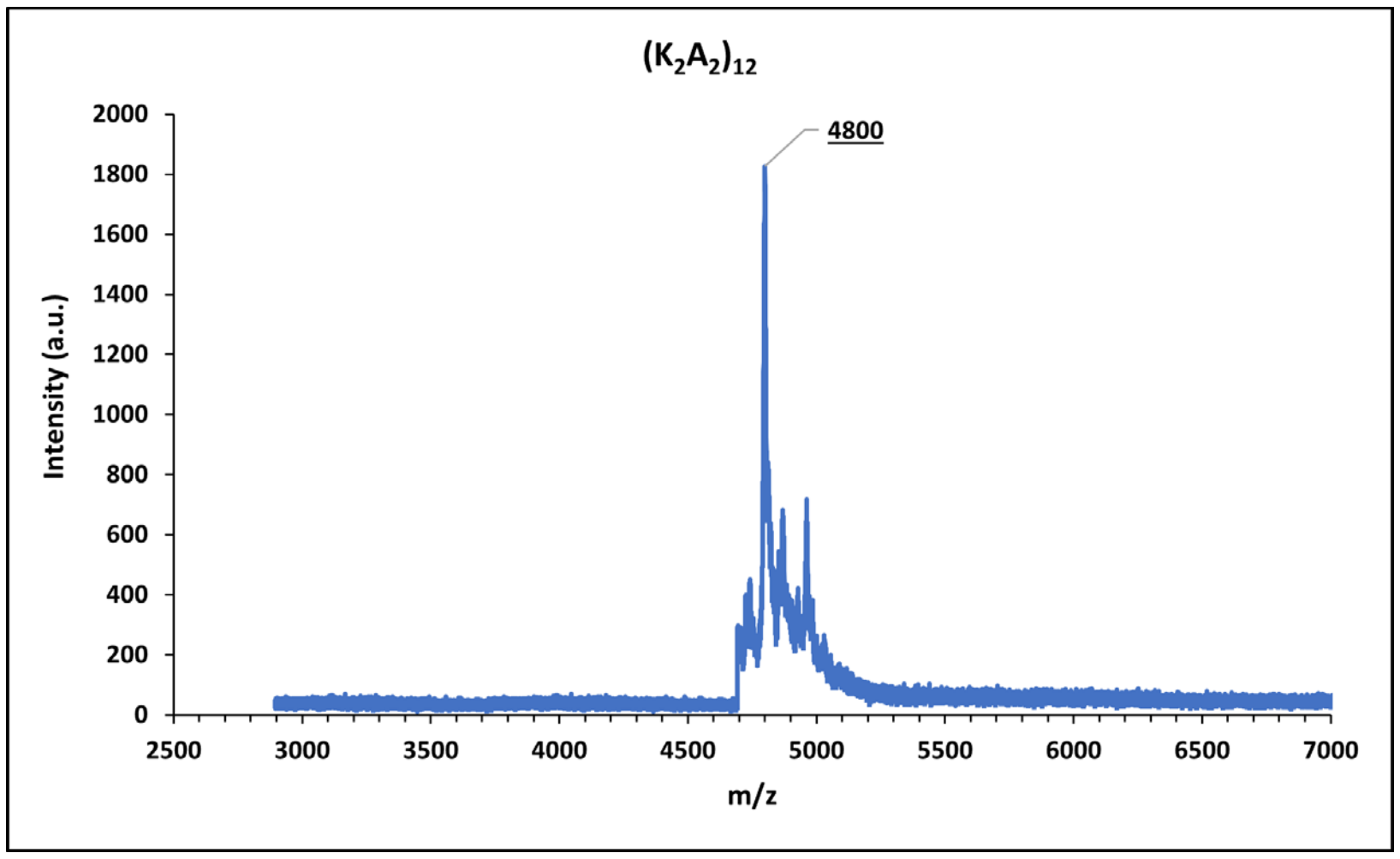

Figure S1f: MALDI-TOF analysis of poly(lysine-co-alanine), $(K_2A_2)_{12}$. The expected theoretical mass is 4799.0 g/mol, excluding counterions. The peak at m/z = 4800 is very close to the expected theoretical mass.

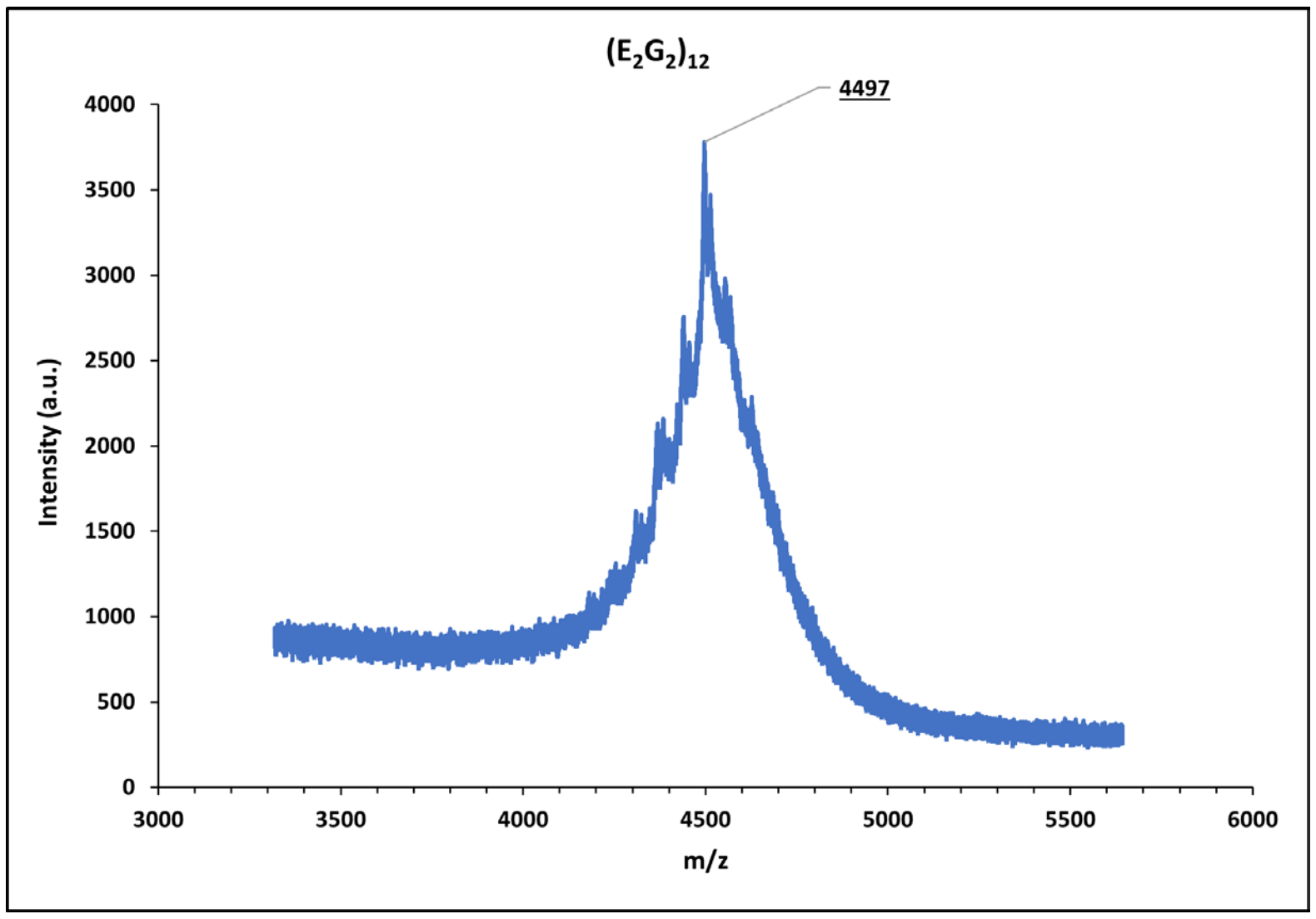

Figure S1h: MALDI-TOF analysis of poly(glutamate-co-glycine), $(E_2G_2)_{12}$. The expected theoretical mass is 4485 g/mol, excluding counterions. The peak at m/z = 4497 is very close to the expected theoretical mass.

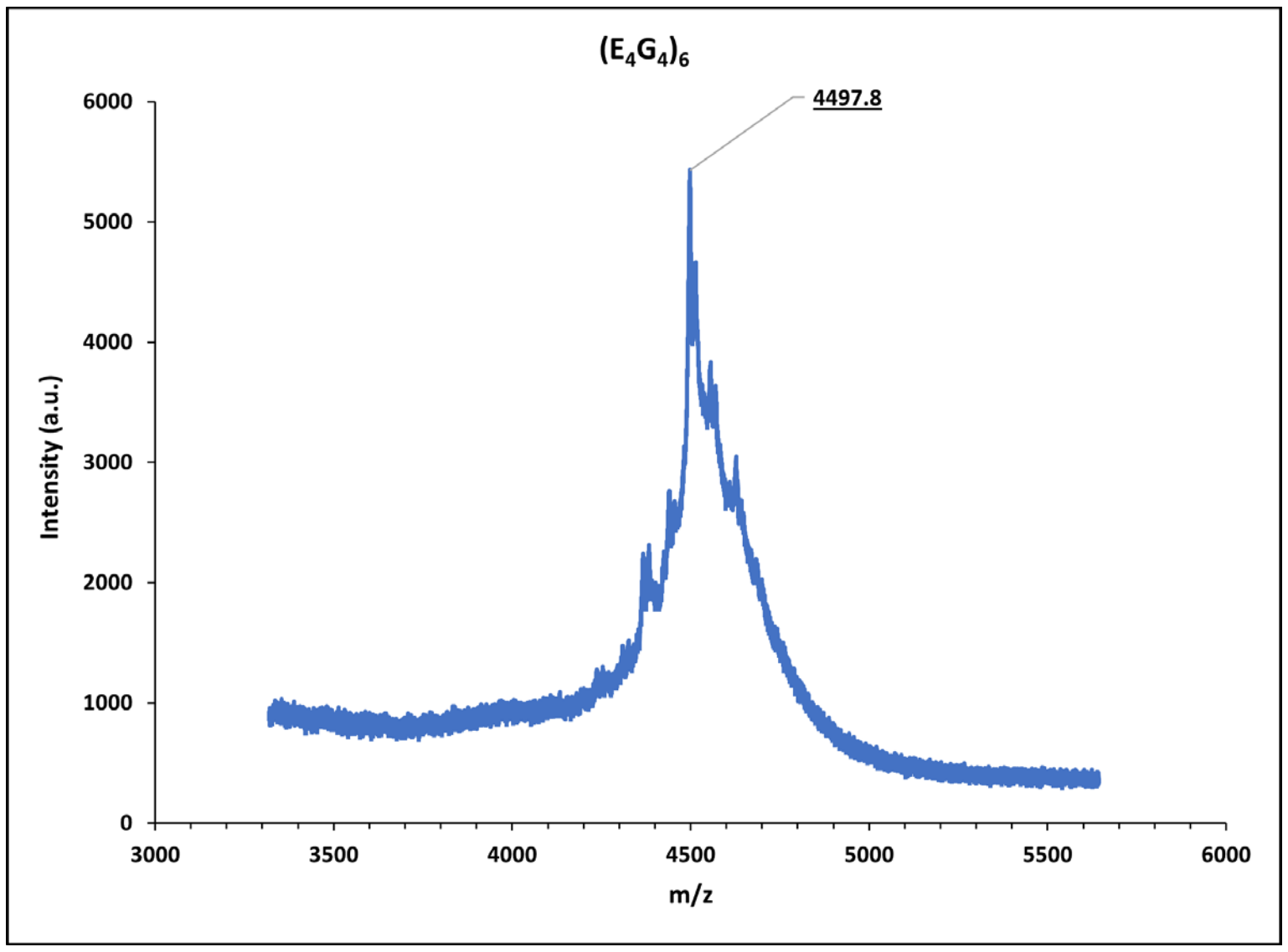

Figure S1i: MALDI-TOF analysis of poly(glutamate-co-glycine), $(E_4G_4)_6$. The expected theoretical mass is 4485 g/mol, excluding counterions. The peak at m/z = 4497.8 is very close to the expected theoretical mass.

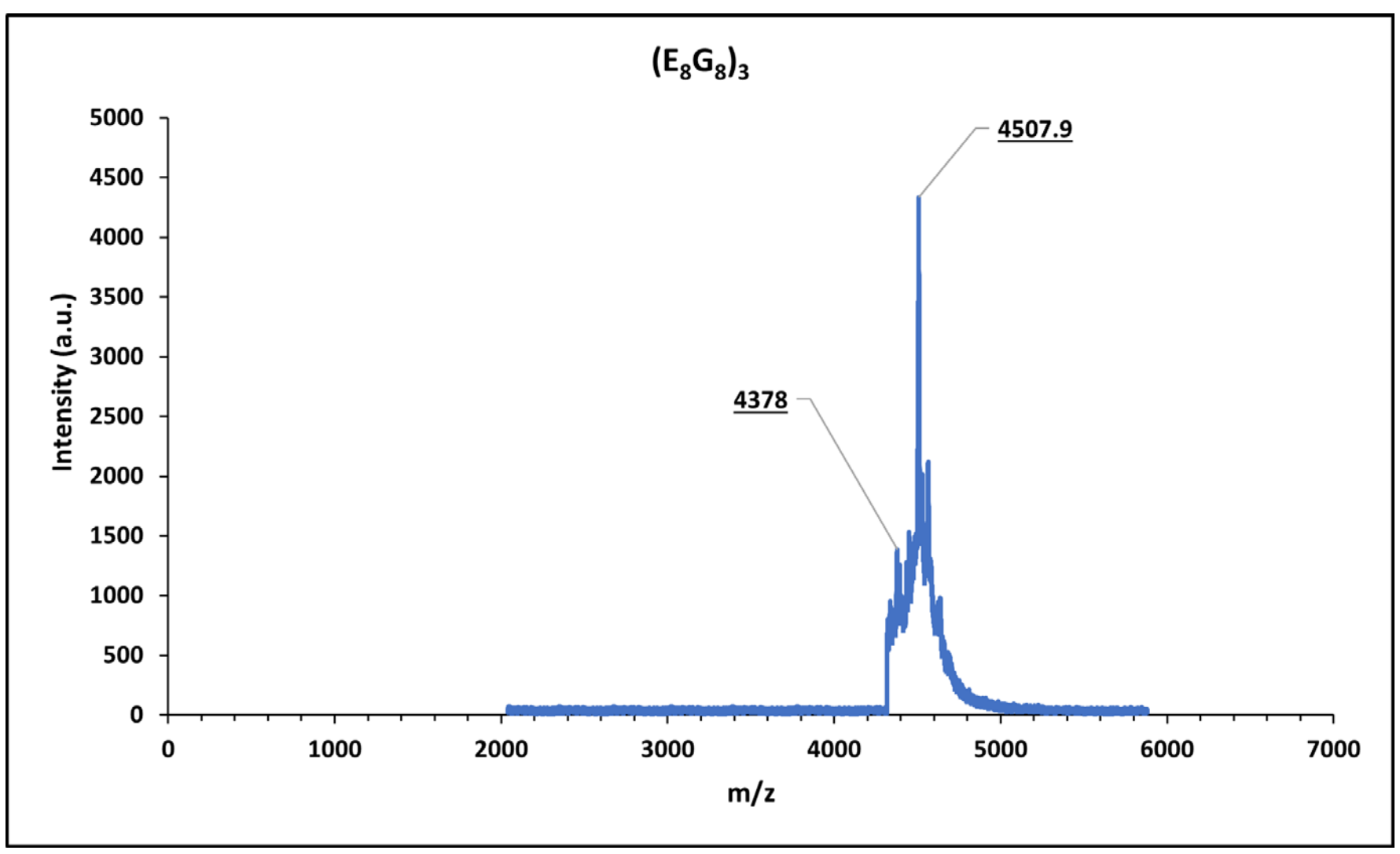

Figure S1j: MALDI-TOF analysis of poly(glutamate-co-glycine), $(E_8G_8)_3$. The expected theoretical mass is 4485 g/mol, excluding counterions. The peak at m/z = 4507.9 could correspond to the presence of a Na^+ counterion. The peak at m/z = 4378 suggests a possible missing glutamate residue.

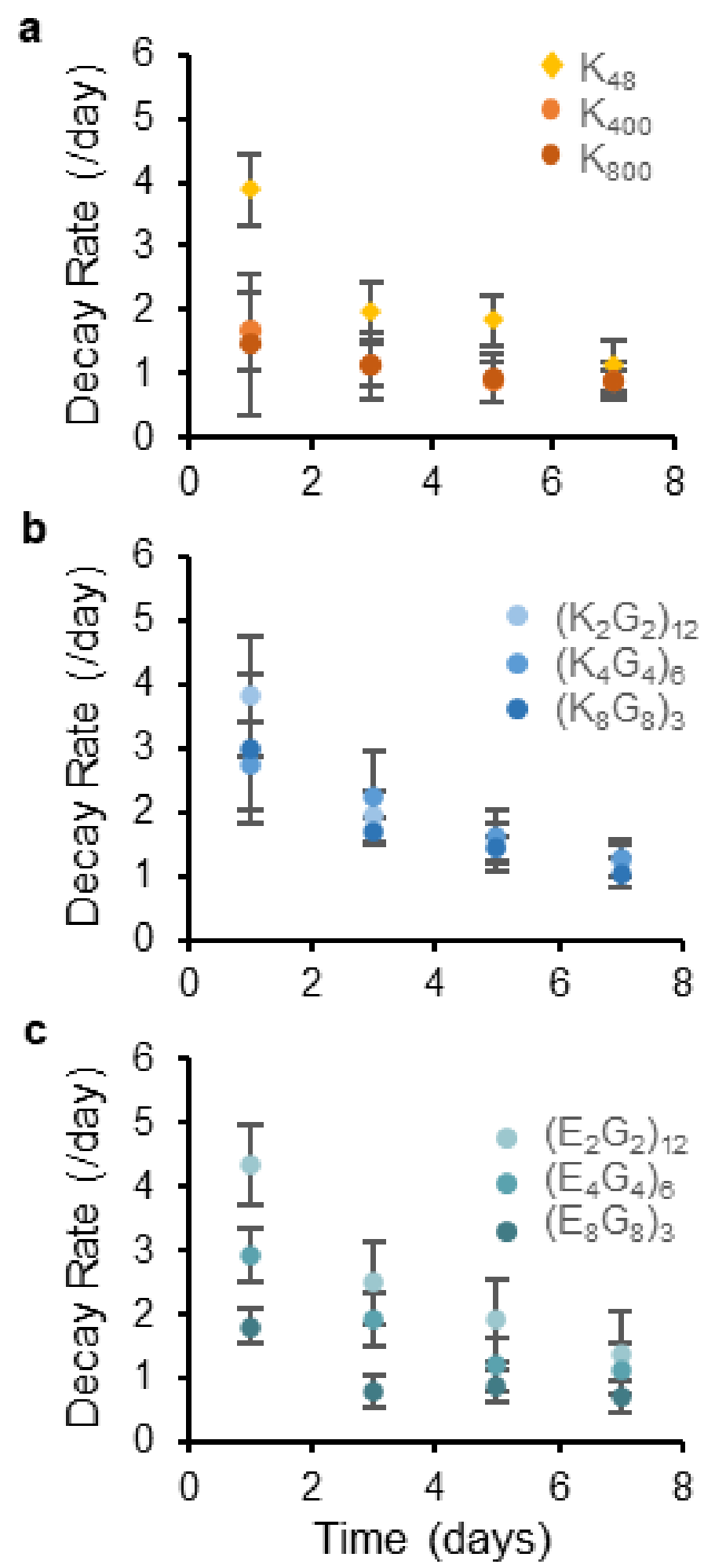

Figure S2: Decay rate for the titer loss of PPV in different coacervate systems, calculated using Eq. 3,4 for the data shown in (a) Figure 3 (b) Figure 4a, and (c) Figure 4b. Data presented are the average of three individual runs of inactivation and the error bars are standard deviations of the decay rates.

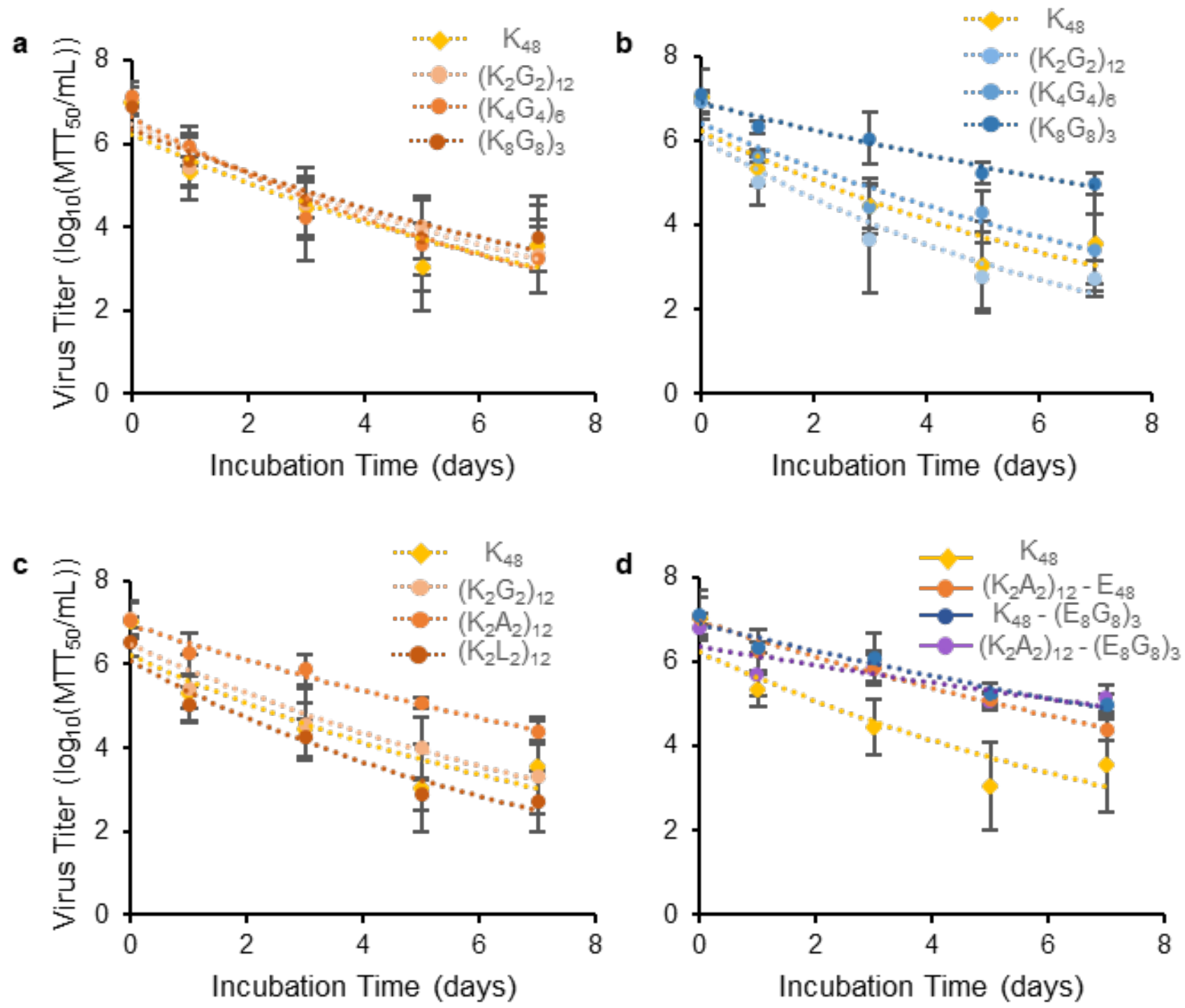

Figure S3: PPV titers post-incubation at 60°C in block-co-polypeptides with neutral glycine blocks: (a) block-co-poly(KG), (b) block-co-poly(EG). (c) PPV titers post-incubation at 60°C in coacervates containing hydrophobic residues. (d) PPV titers post-incubation at 60°C showing a comparison among single patterned polypeptide and double patterned polypeptides. $(K_2A_2)_{12}$ ($\tau = 4$) and $(E_8G_8)_3$ ($\tau = 16$) were used as the patterned polypeptides as the respective systems were the most successful in stabilizing PPV than any other patterned polypeptide system tested. Data presented are the average of three individual runs of inactivation and the error bars are standard deviations in the titers. Dashed lines indicate curve fitting of Eq. 3. LRV data are shown in Figure 4.

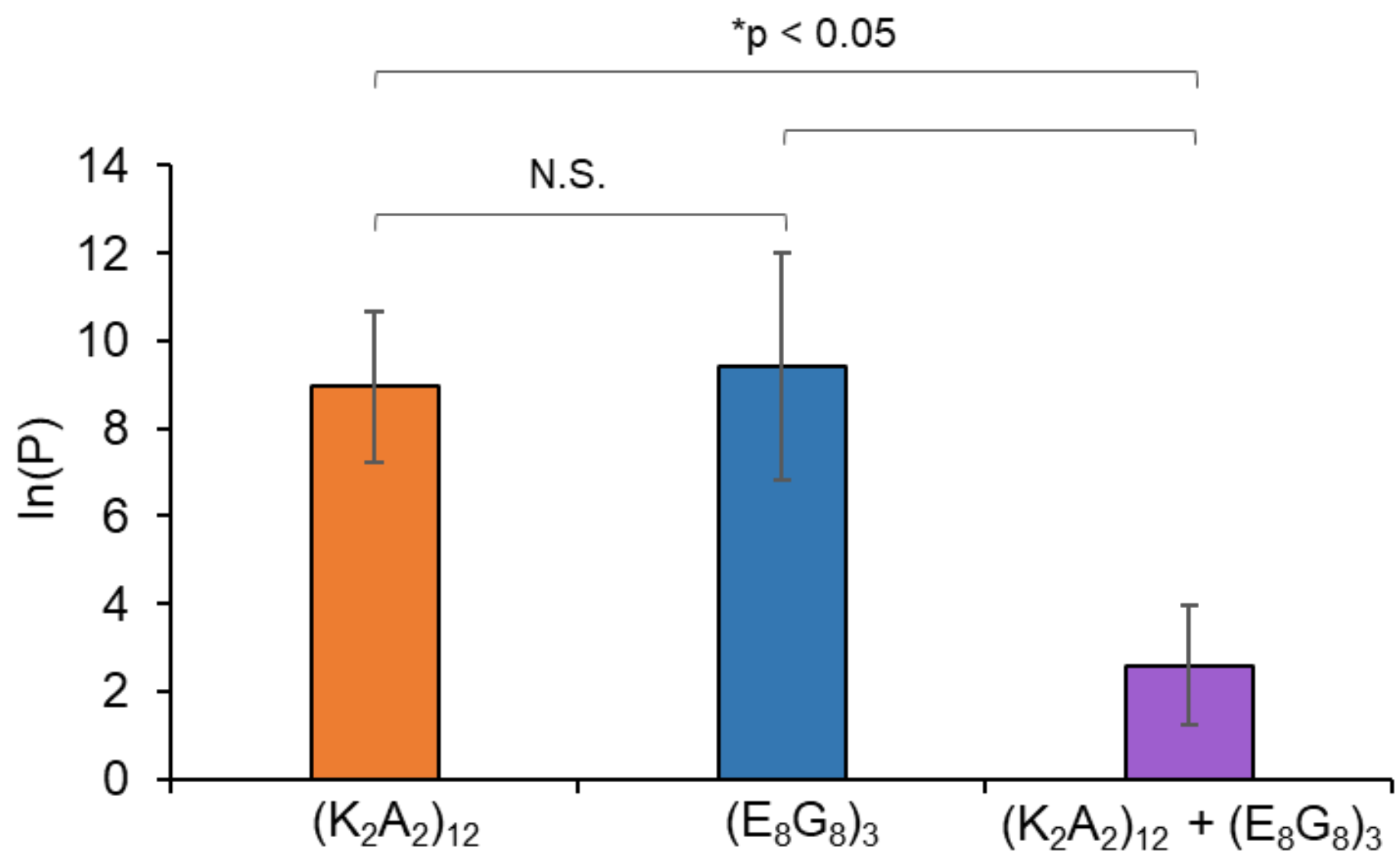

Figure S4: Partitioning of PPV into the coacervate phase, shown as ln(P) showing a comparison among single patterned polypeptide and double patterned polypeptides. $(K_2A_2)_{12}$ ($\tau = 4$) and $(E_8G_8)_3$ ($\tau = 16$) were used as the patterned polypeptides as the respective systems and were the most successful in stabilizing PPV than any other patterned polypeptide system tested. Data presented are the average of three individual runs of inactivation and the error bars are standard deviations in the titers. * $p < 0.05$ using the two-tailed t-test.

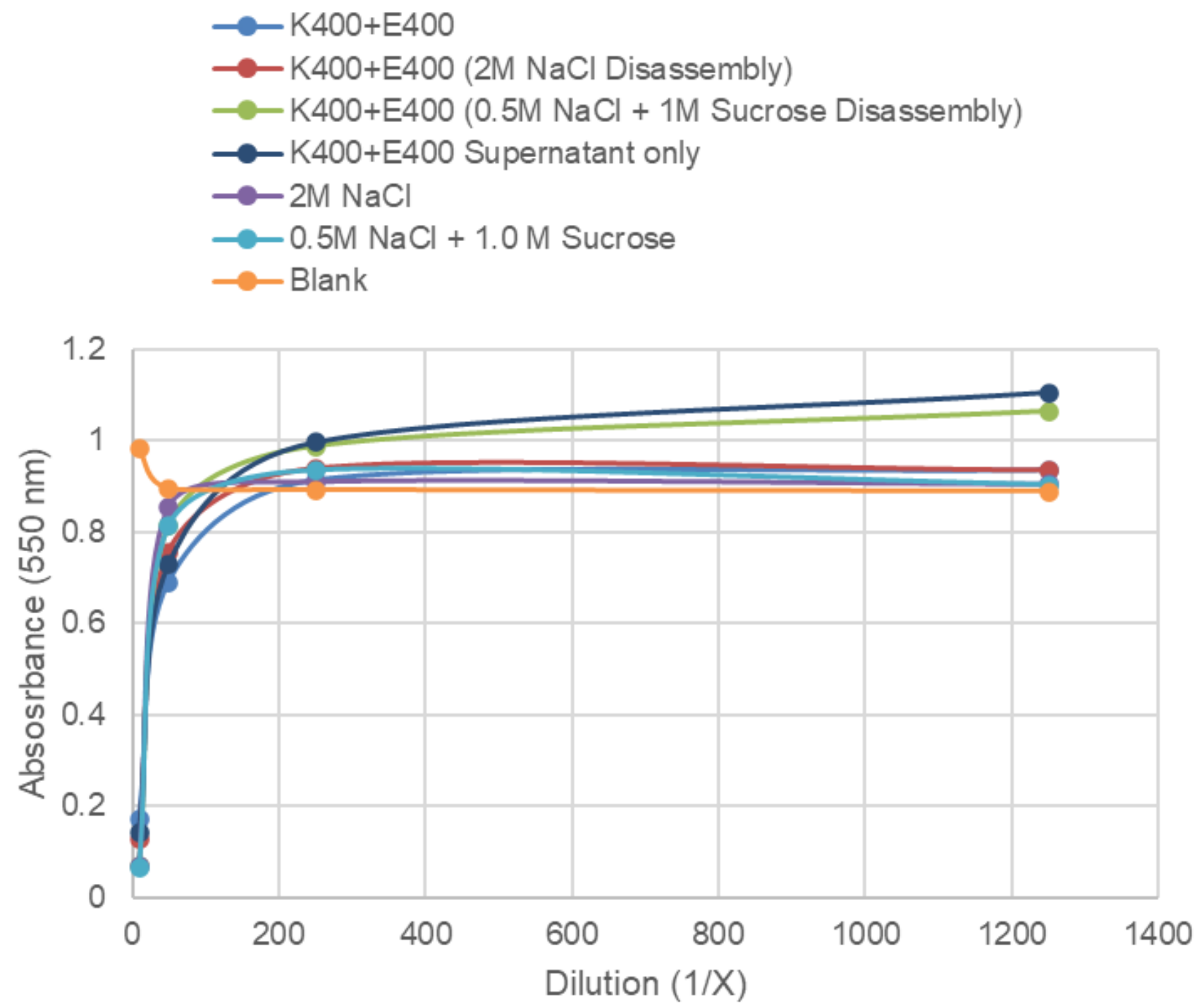

Figure S5: Toxicity of coacervates. Different solutions were added to Vero cells and diluted over four wells. After 24 hours, cell viability was assessed with the MTT solution. All data was conducted in quadruplicate.

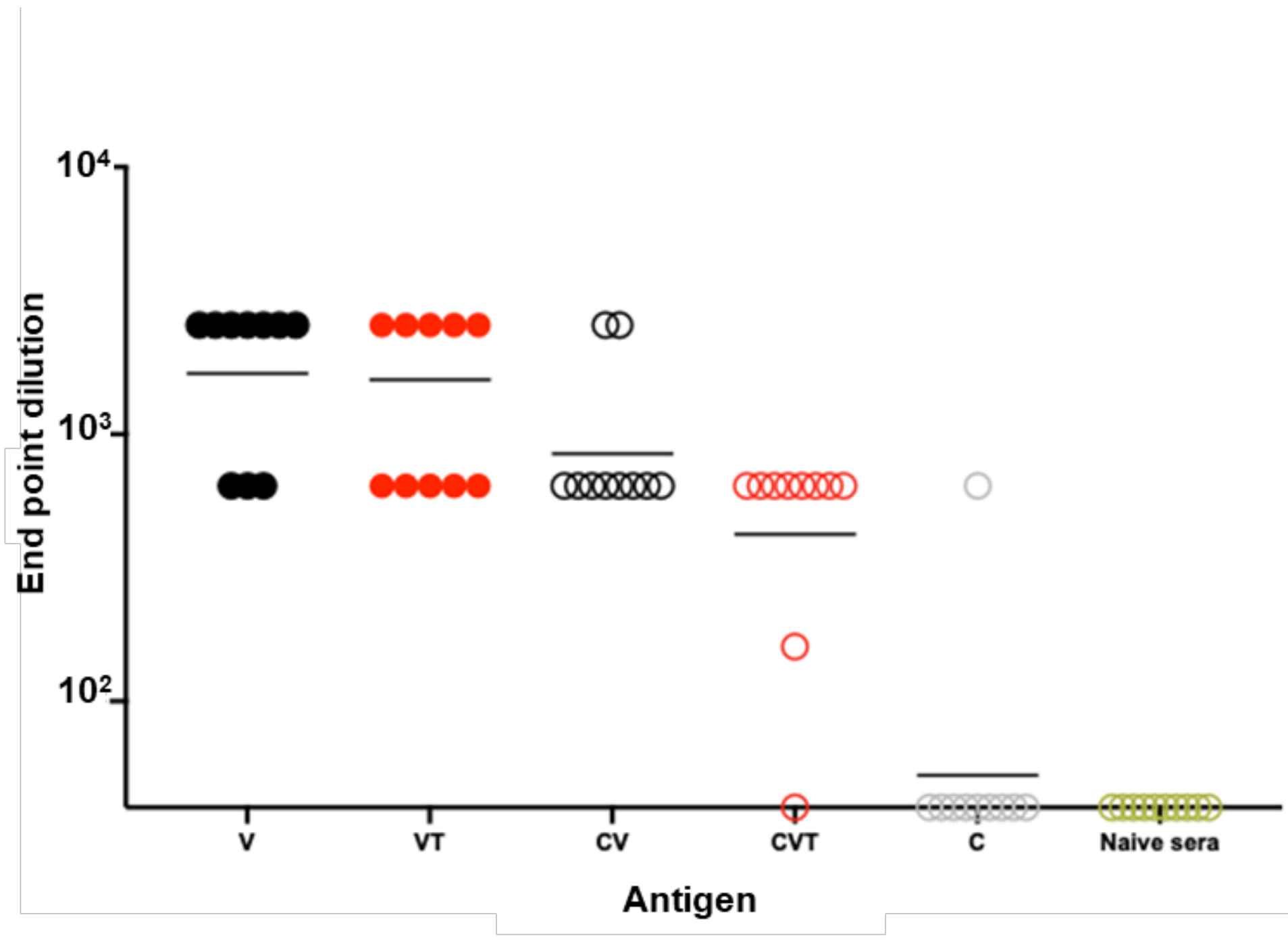

Figure S6: End point dilution for in vivo immunogenicity response. V: virus only stored at 4°C, VT: virus samples after 60°C for 3 days, CV: virus samples in coacervates stored at 4°C, CVT: virus samples in coacervates after 60°C for 3 days, C: coacervate only without virus.

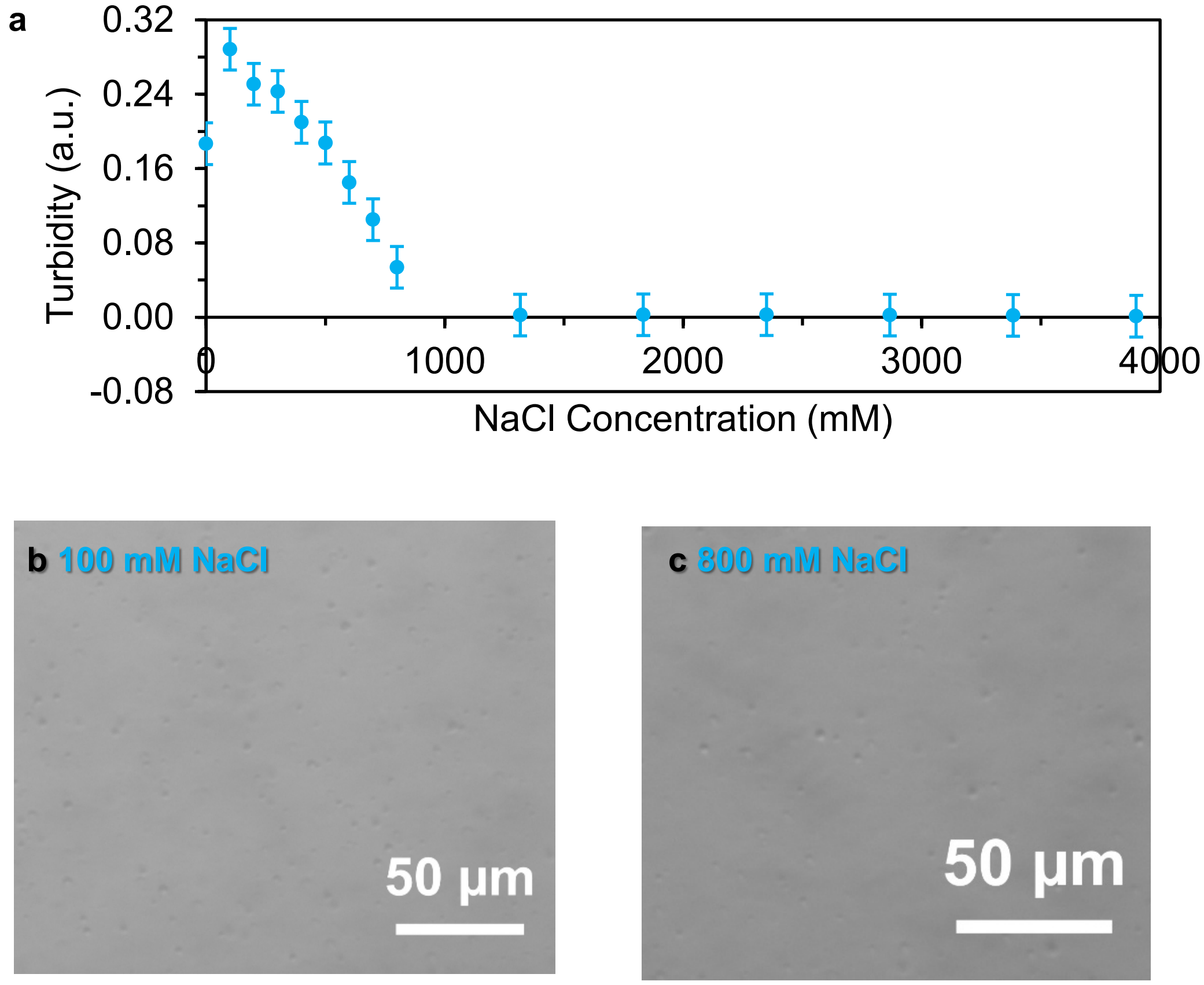

Figure S7: Evaluation of coacervate disassembly with NaCl for the K_{400} - E_{400} system. (a) A plot of turbidity as a function of NaCl concentration and corresponding optical micrographs of samples prepared at (b) 100 mM NaCl, showing droplets, and (c) 800 mM NaCl showing droplets.

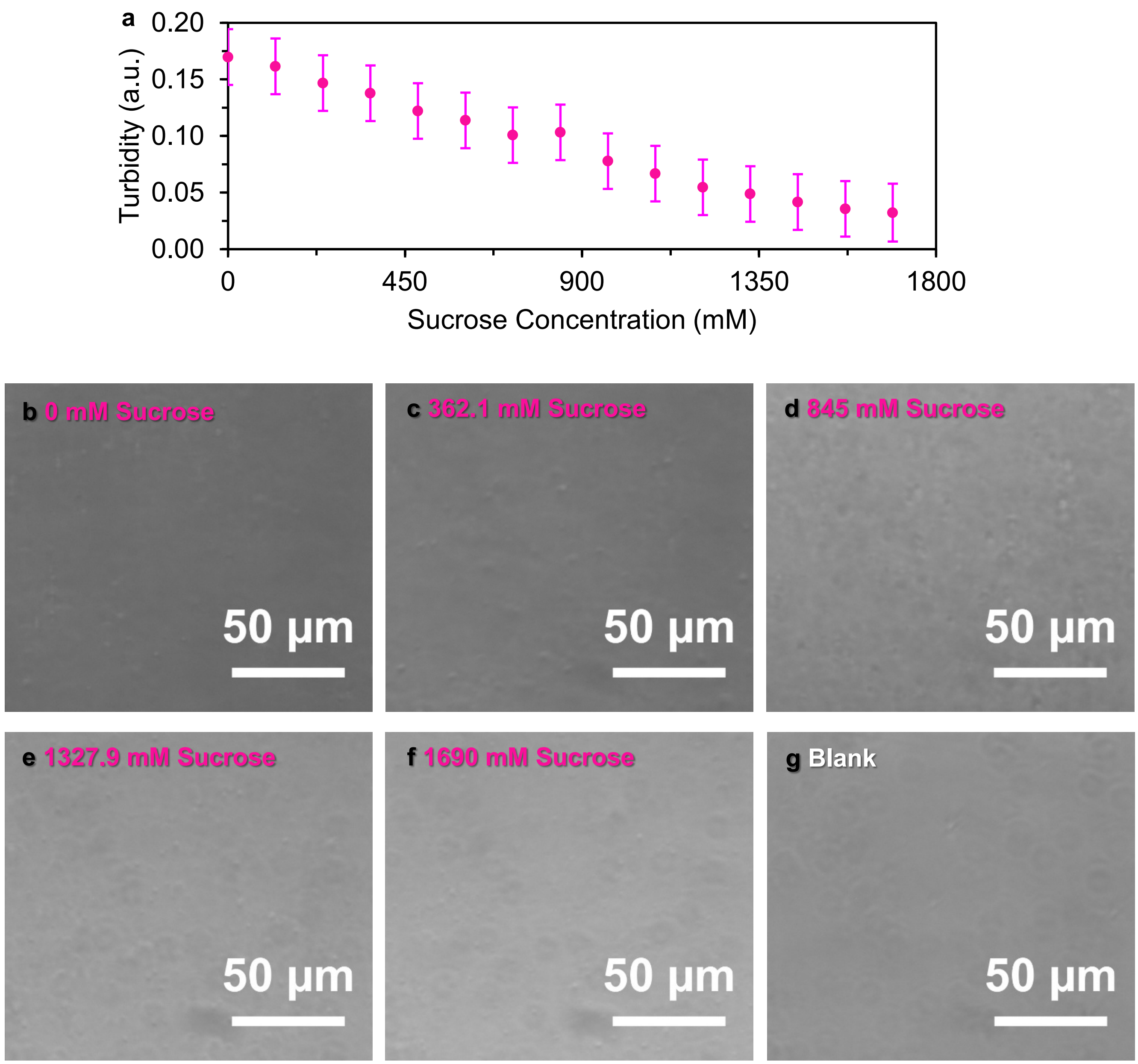

Figure S8: Evaluation of coacervate disassembly with 500 mM NaCl and sucrose for the K_{400} - E_{400} system. (a) A plot of turbidity as a function of sucrose concentration and corresponding optical micrographs of samples prepared at 500 mM NaCl and (b) 0 mM, (c) 362.1 mM, and (d) 845 mM sucrose showing droplets, and (e) 1327.9 mM and (f) 1690 mM sucrose, showing a lack of coacervation, similar to a buffer-only blank (g).
